# Paired In Vitro Susceptibility of Cefiderocol and Colistin in Colistin-Resistant, Extensively Drug-Resistant Gram-Negative Bacilli: A Single-Centre, MIC-Based Analysis of 236 Clinical Isolates

**DOI:** 10.3390/microorganisms14092071

**Published:** 2026-09-16

**Authors:** Elena Hogea, Nicolae Ciprian Pilut, Felix Bratosin, Andra-Nicole Rusnac, Oana Plavitu, Livia Stanga, Horia Silviu Branea

**Affiliations:** 1 Discipline of Microbiology, Victor Babes University of Medicine and Pharmacy, 300041 Timisoara, Romania; hogea.elena@umft.ro (E.H.); stanga.livia@umft.ro (L.S.); 2 Department of Dermatology, Victor Babes University of Medicine and Pharmacy Timisoara, Eftimie Murgu Square 2, 300041 Timisoara, Romania; 3 Center for the Morphologic Study of the Skin (MORPHODERM), Victor Babes University of Medicine and Pharmacy Timisoara, 300041 Timisoara, Romania; 4 Discipline of Infectious Diseases, Victor Babes University of Medicine and Pharmacy, 300041 Timisoara, Romania; 5 Microbiology Laboratory, “Pius Brinzeu” County Clinical Emergency Hospital, 300723 Timisoara, Romania; andra_rusnac@yahoo.com; 6 Clinical Hospital of Infectious Diseases and Pulmonology “Dr. Victor Babes”, 300310 Timisoara, Romania; dr.oanaplavitu@yahoo.com; 7 Department of Internal Medicine I—Medical Semiotics II, Victor Babes University of Medicine and Pharmacy, 300041 Timisoara, Romania; branea.horia@umft.ro

**Keywords:** cefiderocol, colistin, drug resistance, multiple, bacterial, microbial sensitivity tests, Gram-negative bacterial infections

## Abstract

Extensively drug-resistant (XDR) Gram-negative bacilli increasingly exhaust therapeutic options, leaving colistin and cefiderocol as last-resort agents. We assessed whether cefiderocol retains in vitro activity against colistin-resistant isolates, without inferring clinical efficacy. We analysed 236 non-duplicate XDR Gram-negative isolates from 220 patients. Cefiderocol and colistin MICs were determined via reference broth microdilution with EUCAST-required quality control (iron-depleted broth for cefiderocol; *mcr-1*-positive *Escherichia coli* NCTC 13846 for colistin) and interpreted using species-specific EUCAST v16.1 (2026) breakpoints (cefiderocol: Enterobacterales S ≤ 2, I 4, R > 4 mg/L; *Pseudomonas aeruginosa* S ≤ 2, R > 2 mg/L; colistin: *P. aeruginosa* S ≤ 4 mg/L). Because EUCAST publishes no clinical cefiderocol breakpoint for *Acinetobacter baumannii*, the primary analysis comprised species with breakpoints for both agents (*n* = 150; 126 paired); *A. baumannii* is reported as an MIC distribution and the pooled 236-isolate comparison as secondary. In the primary analysis, 120/150 isolates (80.0%) were cefiderocol-susceptible at standard or increased exposure. Among paired isolates, cefiderocol covered 77.0% versus 43.7% for colistin; agreement was negligible (Cohen’s κ = 0.020), with 54 isolates favouring cefiderocol and 12 the reverse (McNemar *p* < 0.001). Conditional in vitro susceptibility among 71 colistin-resistant isolates was 76.1% (95% CI 65.0–84.5), giving 33.3 percentage points of incremental coverage and one additional cefiderocol-susceptible isolate per 2.3 matched isolates tested. Secondary analyses were directionally consistent (incremental coverage 21.8–33.3 points). These in vitro findings support paired testing of both agents to identify isolates retaining cefiderocol activity when colistin does not; clinical benefit was not assessed.

## 1. Introduction

Antimicrobial resistance among Gram-negative bacilli has become one of the most pressing threats in contemporary clinical microbiology. The convergence of carbapenem resistance with resistance to aminoglycosides, fluoroquinolones, and β-lactam/β-lactamase-inhibitor combinations has produced extensively drug-resistant (XDR) phenotypes for which few reliably active agents remain. *Klebsiella pneumoniae*, *Acinetobacter baumannii*, and *Pseudomonas aeruginosa*—the organisms that dominate the present cohort—are ranked by the World Health Organization among the critical-priority pathogens for which new therapeutics are most urgently needed [1]. The XDR designation itself, defined by international consensus as non-susceptibility to at least one agent in all but two or fewer antimicrobial categories, captures precisely the population in which therapeutic choice collapses to one or two remaining drugs [2]. In hospitalised, critically ill populations, infections caused by these organisms carry attributable mortality that frequently exceeds that of the underlying admitting diagnosis, and large prospective cohorts have demonstrated that outcomes vary substantially across regions, even for genetically similar organisms [3].

For much of the past two decades, the polymyxins—chiefly colistin—have served as the pharmacological backbone against carbapenem-resistant Gram-negatives. Their revival, however, reflected desperation rather than pharmacological merit, and the agent required retrospective redevelopment because it entered clinical use before modern pharmacokinetic standards existed [4]. Colistin exhibits unpredictable pharmacokinetics, with plasma concentrations in critically ill patients frequently falling below those required for reliable bacterial killing and negligible penetration into the alveolar compartment [5]. It carries dose-limiting nephrotoxicity and neurotoxicity that constrain escalation [6], and its disposition is further distorted in patients receiving extracorporeal support [7]. Compounding these pharmacological liabilities, colistin resistance has disseminated globally: the discovery of the plasmid-borne *mcr-1* gene demonstrated for the first time that polymyxin resistance could be transferred horizontally [8], and subsequent work has traced *mcr* variants across epidemic plasmid backbones, host species, and continents [9], including wildlife reservoirs [10] and livestock populations in Europe [11]. In *K. pneumoniae* specifically, chromosomal inactivation of the regulatory gene *mgrB* constitutively activates the PhoPQ two-component system, modifying lipid A and conferring colistin resistance that is not only stable but associated with enhanced host-to-host transmission [12].

Cefiderocol represents a mechanistically distinct alternative. As a siderophore cephalosporin, it chelates extracellular ferric iron and exploits the bacterial iron-transport machinery to achieve periplasmic entry through a “Trojan horse” strategy, circumventing the porin loss and efflux upregulation that defeat conventional β-lactams [13,14]. It demonstrates in vitro stability to enzymes drawn from each of the four Ambler classes, including many serine- and metallo-carbapenemases, but this stability is neither uniform nor absolute: activity is reduced or lost against particular enzyme variants, notably certain NDM and PER variants and some AmpC and extended-spectrum β-lactamases, and enzyme-independent mechanisms further modulate susceptibility. It, therefore, retains activity against many, but not all, isolates for which no other β-lactam remains viable; described resistance mechanisms centre not on porins or efflux but on mutations in iron transporters, AmpC omega-loop alterations, and a restricted set of β-lactamases [15]. Its clinical development followed an unusual pathogen-focused regulatory pathway rather than the conventional site-of-infection route [16], and the pivotal CREDIBLE-CR programme, in which an imbalance in all-cause mortality was observed relative to best available therapy, continues to shape cautious positioning of the drug in practice [17]. This mechanistic distinctness from the polymyxin target—the lipopolysaccharide of the outer membrane—provides the biological rationale for the central hypothesis of this study.

A substantial body of surveillance data now describes cefiderocol activity. The multinational SIDERO-WT programme, spanning five consecutive annual collections from North America and Europe, reported susceptibility exceeding 99% for Enterobacterales and *P. aeruginosa* and 96% for *A. baumannii* complex [18]; comparable figures emerged from the SENTRY programme, in which cefiderocol was the most active agent tested against XDR *P. aeruginosa* [19]. National datasets from England [20] and Japan [21] reproduced these findings across differing carbapenemase epidemiologies, and focused analyses confirmed retained activity against metallo-β-lactamase producers [22] and across defined β-lactamase carriage profiles [23]. Yet a systematic review and meta-analysis of 78 studies and more than 82,000 isolates tempered this optimism, showing that although cefiderocol non-susceptibility is low overall, it rises to 12–13% among carbapenem-resistant Enterobacterales and carbapenem-resistant *A. baumannii* and approaches 39–45% in New Delhi metallo-β-lactamase producers, with estimates varying considerably by breakpoint definition and by single-centre setting [24].

Despite this research, comparatively little attention has been paid to the specific question that arises most often at the bedside: when colistin has failed or cannot be used, does cefiderocol offer a genuine alternative for that same isolate? Aggregate susceptibility percentages, reported drug by drug, cannot answer this question because they treat each agent’s activity as if it were independent of the other. A small single-centre study of 101 carbapenem-resistant isolates observed that all colistin-resistant organisms remained cefiderocol-susceptible, but with only six colistin-resistant isolates, the estimate was too imprecise to guide practice [25]. Narrative reviews of carbapenem-resistant *A. baumannii* management have likewise called for data on cefiderocol’s position relative to other salvage options [26], and current IDSA guidance positions cefiderocol largely as a later-line agent, reflecting the absence of robust comparative microbiological evidence [27]. What is required instead is a paired, within-isolate analysis that quantifies discordance and, in particular, the conditional probability of cefiderocol susceptibility given colistin resistance. To our knowledge, few studies have quantified this conditional susceptibility in a large single-centre XDR collection using reference broth microdilution for both agents, which is the specific gap the present analysis addresses.

Accordingly, the present study was designed around a within-isolate comparative framework. Rather than reporting two parallel antibiograms, we treated each isolate as its own control and examined the joint distribution of cefiderocol and colistin phenotypes. This design permits the calculation of categorical agreement statistics, directional discordance testing, and the conditional in vitro susceptibility of cefiderocol given colistin resistance—the proportion of colistin-resistant isolates that remain cefiderocol-susceptible—together with the incremental in vitro coverage and incremental diagnostic yield that follow from it. These are laboratory metrics describing what paired testing adds to the antibiogram; they are not measures of clinical benefit. The objectives of this study were, therefore, three-fold: first, to characterise cefiderocol activity across a real-world collection of 236 XDR Gram-negative isolates spanning five specimen types; second, to formally quantify the concordance and discordance between cefiderocol and colistin using paired statistics; and third, to test whether cefiderocol retains meaningful activity against colistin-resistant organisms and to identify the microbiological and demographic factors that independently predict cefiderocol resistance, thereby informing the composition of last-line susceptibility testing panels. Because treatment and outcome data were not available, this study cannot address therapeutic efficacy.

## 2. Materials and Methods

### 2.1. Study Design, Setting, and Bacterial Isolates

This was a retrospective, single-centre, laboratory-based observational study conducted on consecutive XDR Gram-negative bacilli recovered from clinical specimens processed by the microbiology laboratory over the surveillance interval spanning October 2025 to June 2026. A total of 236 non-duplicate isolates were included. Specimens were distributed across five source categories: lower respiratory tract material (sputum and bronchial aspirates; n = 121), urine (n = 45), purulent secretions (n = 30), intravascular and bronchial catheter tips (n = 30), and blood cultures (n = 10). Only the first isolate of a given species from a given patient within the study window was retained in order to avoid duplication bias. Because de-duplication was applied per species rather than per patient, a patient carrying more than one XDR species could contribute more than one isolate. The 236 isolates originated from 220 unique patients: 205 patients contributed a single isolate, 14 contributed two isolates of different species, and 1 contributed three. The 16 isolates beyond the first per patient (*K. pneumoniae*, n = 8; *A. baumannii*, n = 5; *P. aeruginosa*, n = 3), therefore, represent 6.8% of the collection, and the independence assumption underlying the paired and regression analyses is violated only for this small fraction; because each of these isolates is a different species from the same patient, the within-patient correlation of cefiderocol and colistin phenotypes is expected to be weak. To confirm that this had no material effect, a patient-level sensitivity analysis restricted to the first isolate per patient and a repetition of the primary regression model with cluster-robust standard errors clustered on patient were performed and are reported in Section 3.4. Susceptibility was assessed across the antimicrobial categories relevant to each organism, and XDR was operationally defined as non-susceptibility to at least one agent in all but two or fewer of these applicable categories. The antimicrobial categories and representative agents used for XDR classification, together with the category-level resistance frequencies observed in the cohort, are summarised in Table 1, and the categories applicable to each species, together with the species-specific denominators used when the XDR definition was applied, are given in Supplementary Table S1. Every antimicrobial category was interpreted with EUCAST v16.1 (2026) clinical breakpoints for the species tested. Where the international consensus category list requires an agent for which EUCAST publishes no breakpoint for that species—extended-spectrum cephalosporins, piperacillin–tazobactam, tigecycline, ceftazidime–avibactam and aztreonam–avibactam in *A. baumannii*, and trimethoprim–sulfamethoxazole, tigecycline and aztreonam–avibactam in *P. aeruginosa*—results were generated for completeness but were not scored towards the XDR definition, so that no category lacking a EUCAST breakpoint contributed to the classification of any isolate. For *A. baumannii,* this leaves four scored categories (carbapenems, fluoroquinolones, aminoglycosides and folate-pathway inhibitors) once the two study agents are set aside; all 83 *A. baumannii* isolates met the XDR definition on these EUCAST-breakpoint categories alone, so no isolate entered the collection on the strength of a category without a breakpoint. The specific agents tested, the test method used for each and the breakpoint source applied are set out by species in Supplementary Table S2. Organisms were included solely on the basis of meeting the XDR definition, applied according to the international consensus criteria of non-susceptibility to at least one agent in all but two or fewer antimicrobial categories [2].

Species identification followed standard laboratory workflow, and the collection comprised *Klebsiella pneumoniae* (n = 121, 51.3%), *Acinetobacter baumannii* (n = 83, 35.2%), *Pseudomonas aeruginosa* (n = 25, 10.6%), *Escherichia coli* (n = 4), and *Escherichia vulneris* (n = 3). Available patient descriptors were limited to age and biological sex; the cohort had a median age of 69 years (interquartile range 60–75, full range 24–95) and a male predominance (150 males, 63.6%). Because the dataset was fully de-identified and generated as part of routine diagnostic activity, the analysis carried the ethical profile of a laboratory surveillance audit. No patient-level clinical outcomes, comorbidity data, or treatment histories were available, and the study was, therefore, confined to microbiological endpoints throughout.

**Table 1:** Antimicrobial categories and representative agents used to define the extensively drug-resistant (XDR) phenotype, with category-level non-susceptibility frequencies observed in the cohort (n = 236).

Antimicrobial Category	Representative Agent(s) Tested	Non-Susceptible, n/N (%)
Antipseudomonal carbapenems	Meropenem, imipenem	236/236 (100)
Extended-spectrum cephalosporins	Ceftazidime, cefepime	234/236 (99.2)
Antipseudomonal fluoroquinolones	Ciprofloxacin, levofloxacin	228/236 (96.6)
Antipseudomonal penicillins + BLI	Piperacillin-tazobactam	231/236 (97.9)
Aminoglycosides	Gentamicin, amikacin, tobramycin	210/236 (89.0)
Folate-pathway inhibitors	Trimethoprim-sulfamethoxazole	205/236 (86.9)
Monobactam/BLI combination	Aztreonam-avibactam	209/236 (88.6)
Cephalosporin/BLI combination	Ceftazidime-avibactam	190/236 (80.5)
Glycylcyclines/tetracyclines	Tigecycline (where applicable)	150/236 (63.6)
Polymyxins	Colistin	86/211 (40.8)
Siderophore cephalosporin	Cefiderocol	42/236 (17.8)

Categories follow the international consensus definition of Magiorakos et al. [2]; only categories applicable to each organism were counted, and intrinsic resistance was not scored as acquired non-susceptibility. XDR was operationally defined as non-susceptibility to at least one agent in all but two or fewer applicable categories. Denominators in Table 1 refer to all isolates tested against the agents in that category; the subset of categories that was actually scored towards the XDR definition differed by species because intrinsically resistant species–agent combinations were not counted, and these species-specific denominators are set out in Supplementary Table S1. Colistin and cefiderocol are shown for completeness; their MIC-based results form the basis of the paired analysis. BLI, beta-lactamase inhibitor. Colistin non-susceptibility for *P. aeruginosa* is scored against the EUCAST parenthetical breakpoint of >4 mg/L and cefiderocol non-susceptibility for Enterobacterales against >4 mg/L, the intermediate (susceptible, increased exposure) category at 4 mg/L not being counted as non-susceptible; the breakpoints applied to every species–agent combination are given in Table 2. All categories were interpreted with EUCAST v16.1 (2026) breakpoints, and any species–agent combination for which EUCAST publishes no breakpoint was excluded from the XDR calculation for that species (Supplementary Tables S1 and S2).

**Table 2:** Species-specific EUCAST v16.1 (2026) clinical breakpoints applied to cefiderocol and colistin in this study.

Agent	Species or Group	Susceptible (S)	Susceptible, Increased Exposure (I)	Resistant (R)	Breakpoint Source
Cefiderocol	Enterobacterales (*K. pneumoniae*, *E. coli*)	MIC ≤ 2 mg/L	MIC = 4 mg/L	MIC > 4 mg/L	EUCAST v16.1 (2026)
Cefiderocol	*P. aeruginosa*	MIC ≤ 2 mg/L	No I category	MIC > 2 mg/L	EUCAST v16.1 (2026)
Cefiderocol	*A. baumannii*	No clinical breakpoint ^‡^	—	No clinical breakpoint ^‡^	EUCAST v16.1 (2026)
Cefiderocol	*E. vulneris*	No species-specific breakpoint ^§^	—	No species-specific breakpoint ^§^	—
Colistin	Enterobacterales (*K. pneumoniae*, *E. coli*)	MIC ≤ 2 mg/L	No I category	MIC > 2 mg/L	EUCAST v16.1 (2026)
Colistin	*A. baumannii*	MIC ≤ 2 mg/L	No I category	MIC > 2 mg/L	EUCAST v16.1 (2026)
Colistin	*P. aeruginosa*	MIC ≤ 4 mg/L	No I category	MIC > 4 mg/L	EUCAST v16.1 (2026), breakpoint listed in parentheses ^†^
Colistin	*E. vulneris*	No species-specific breakpoint ^§^	—	No species-specific breakpoint ^§^	—

^‡^ EUCAST publishes no clinical cefiderocol breakpoint for *A. baumannii*. It provides instead a graded interpretation in which an MIC of ≤0.5 mg/L, an MIC of 1–2 mg/L and an MIC of >2 mg/L are supported by different levels of evidence, and it does not recommend a simple susceptible-or-resistant classification. Accordingly, no categorical assignment was made for this species, and its cefiderocol results are presented as an MIC distribution against those three evidence tiers (Section 3.4). ^§^ No species-specific breakpoint exists for *E. vulneris* for either agent; its results are reported descriptively only. ^†^ For colistin and *P. aeruginosa*, EUCAST lists the breakpoint in parentheses, indicating a breakpoint for which the evidence is limited; results interpreted against it are flagged accordingly throughout. Isolates in the I category are reported separately and, where a dichotomous grouping is required, are grouped with susceptible isolates; susceptible-only (S) proportions are given alongside. MIC, minimum inhibitory concentration.

### 2.2. Antimicrobial Susceptibility Testing

Susceptibility testing was performed and interpreted in accordance with current European Committee on Antimicrobial Susceptibility Testing (EUCAST) standards, using both qualitative and quantitative methods. Qualitative screening employed the Kirby–Bauer disc diffusion technique on Mueller–Hinton agar. A 0.5 McFarland inoculum (approximately 10^8^ CFU/mL), prepared by emulsifying two to three colonies from a pure culture in physiological saline, was lawn-inoculated in three directions with a final circumferential streak. Antimicrobial-impregnated discs—including cefiderocol (30 µg), amikacin (30 µg), gentamicin (10 µg), aztreonam/avibactam, ceftazidime/avibactam, and meropenem—were applied at appropriate spacing from one another and from the plate margin. Plates were incubated at 37 °C for 24 h, after which inhibition-zone diameters were measured and categorised as susceptible, intermediate, or resistant against the species-specific EUCAST tables. Disc diffusion was used only as a preliminary screening step and did not contribute to isolate inclusion or to the final susceptibility categorisation, which rested exclusively on broth microdilution MICs; where the disc and MIC categories diverged for cefiderocol, the MIC result was taken as definitive.

The definitive quantitative endpoints used throughout this analysis were minimum inhibitory concentrations (MICs) determined by broth microdilution, which is the reference method for both cefiderocol and colistin; gradient diffusion and automated alternatives are well documented to produce unacceptable very-major-error rates for the polymyxins and are not recommended for this purpose [28,29]. Cefiderocol MICs were obtained using Bruker UMIC cefiderocol strips (Bruker Daltonik GmbH, Bremen, Germany) in iron-depleted cation-adjusted Mueller–Hinton broth—iron depletion being essential to avoid falsely low MICs, given the drug’s iron-dependent uptake—while colistin MICs were determined by Bruker UMIC Colistin (Bruker Daltonik GmbH, Bremen, Germany) in Mueller–Hinton II broth. From a 0.5 McFarland suspension, 25 µL was transferred into the 5 mL broth vial, and 100 µL was dispensed into each of the twelve wells spanning 0.03–32 µg/mL. After resealing and incubation at 35 ± 1 °C for 18 ± 2 h in a humidified UMIC chamber, the MIC was read as the lowest concentration showing no visible growth, turbidity or button formation at the well base being taken as growth. MICs were interpreted with the species-specific clinical breakpoints of EUCAST Breakpoint Table v16.1 (2026), which differ by species and are set out in full in Table 2; a single uniform threshold was not applied. For cefiderocol against Enterobacterales, the breakpoints are susceptible at MIC ≤ 2 mg/L, susceptible at increased exposure (I) at MIC = 4 mg/L, and resistant at MIC > 4 mg/L, so isolates with an MIC of exactly 4 mg/L were categorised as I and not as resistant; for cefiderocol against *P. aeruginosa,* the breakpoints are susceptible ≤ 2 mg/L and resistant > 2 mg/L, with no I category. For colistin, the breakpoints are susceptible ≤ 2 mg/L and resistant > 2 mg/L for Enterobacterales and for *A. baumannii*, whereas for *P. aeruginosa,* EUCAST lists the breakpoint in parentheses at susceptible ≤ 4 mg/L and resistant > 4 mg/L; the parenthetical *P. aeruginosa* value was, therefore, used for that species, and results interpreted against it are flagged as resting on a breakpoint that EUCAST itself qualifies. EUCAST publishes no clinical cefiderocol breakpoint for *A. baumannii*, offering instead a graded interpretation in which MIC ≤ 0.5 mg/L, 1–2 mg/L and >2 mg/L carry different levels of evidence; no susceptible-or-resistant category was, therefore, assigned to *A. baumannii*, and its cefiderocol results are reported only as an MIC distribution against those evidence tiers. *E. coli* followed *Enterobacterales criteria*; *E. vulneris*, for which no species-specific EUCAST breakpoint exists, is likewise reported as an MIC distribution without categorical assignment. Because categorical interpretation is not valid in the absence of a species-specific clinical breakpoint, the primary analysis of this study is restricted to the breakpoint-valid subpopulation, comprising those species–agent combinations for which EUCAST publishes species-specific clinical breakpoints for both cefiderocol and colistin, namely *K. pneumoniae*, *E. coli* and *P. aeruginosa* (n = 150 isolates, of which 126 had an interpretable paired colistin MIC) (Section 3.1). Cefiderocol results for *A. baumannii* and *E. vulneris* are presented as complete MIC distributions without categorical assignment (Section 3.4). Where categorical cefiderocol results for *A. baumannii* and *E. vulneris* appear in the pooled tables, they are descriptive classifications made against the PK/PD (non-species-related) value of 2 mg/L, are labelled as such, belong to the secondary descriptive analysis of the full collection, and must not be read as clinical susceptibility categories.

Quality control was performed on every day of testing and with every new lot of panels, in accordance with EUCAST and CLSI recommendations; the control strains, media and acceptance ranges used are given in Table 3. For cefiderocol, iron-depleted cation-adjusted Mueller–Hinton broth (ID-CAMHB) was used for the control strains as well as for the clinical isolates, and adequate iron depletion was verified on each testing day by confirming that the cefiderocol MIC for *Escherichia coli* ATCC 25922 fell within the 0.03–0.25 mg/L acceptance range and that for *Pseudomonas aeruginosa* ATCC 27853 within 0.06–0.5 mg/L; a control MIC above its acceptance range would indicate insufficient iron depletion and invalidate the run, and no testing was performed in iron-replete broth. For colistin, EUCAST requires an *mcr-1*-positive control in addition to a susceptible control strain for broth microdilution: *E. coli* ATCC 25922 (acceptance range 0.25–2 mg/L) and *P. aeruginosa* ATCC 27853 (0.5–4 mg/L) served as the susceptible controls, and the *mcr-1*-positive *E. coli* NCTC 13846 was included with every panel lot, its expected MIC being 4 mg/L (acceptance range 2–4 mg/L). In line with EUCAST requirements for polymyxin broth microdilution, colistin sulfate rather than colistimethate sodium was used, no polysorbate-80 or other surfactant was added, and plain untreated polystyrene trays were used. Across 240 quality-control determinations, 236 (98.3%) fell within the acceptance range; the 4 out-of-range results were each one doubling dilution outside the range, and all clinical isolates tested in the affected runs were repeated once the control had returned to range.

### 2.3. Definition of Study Groups and Comparison Framework

The analytic architecture was built on a paired, within-isolate comparison of the two last-resort agents. The primary categorical grouping variable was the cefiderocol phenotype, dichotomised as resistant versus not resistant, against which demographic, microbiological, and specimen characteristics were compared in order to identify correlates of resistance. Isolates falling in the susceptible-at-increased-exposure (I) category, which arises only for cefiderocol against Enterobacterales at an MIC of 4 mg/L, were grouped with susceptible isolates for this dichotomy, because I denotes an organism that remains treatable with an increased dosing regimen rather than one against which the agent has failed. The number of I isolates is reported separately wherever the grouping is used, and susceptible-only (S) proportions are given alongside, so that the effect of this convention on every estimate is visible. In parallel, each isolate’s colistin phenotype was retained so that the two agents could be cross-tabulated within the same isolate rather than compared as independent aggregate rates. This yielded a four-cell joint distribution (cefiderocol-S/colistin-S, S/R, R/S, R/R) that formed the substrate for all concordance and discordance analyses reported below. The population in which these analyses are primary is the breakpoint-valid subpopulation defined in Section 2.2, comprising only those species–agent combinations for which EUCAST publishes species-specific clinical breakpoints for both agents (*K. pneumoniae*, *E. coli* and *P. aeruginosa*). The corresponding analyses in the full 236-isolate collection, which necessarily assign cefiderocol categories to *A. baumannii* and *E. vulneris* on a PK/PD (non-species-related) basis, are retained as secondary descriptive analyses and are labelled as such wherever they appear.

The principal comparison of clinical interest—and the source of this study’s intended novelty—was the conditional subgroup of colistin-resistant isolates, within which the proportion remaining cefiderocol-susceptible was designated the conditional in vitro susceptibility of cefiderocol given colistin resistance. The term “rescue rate” is deliberately avoided, because no treatment, exposure or outcome data were available, and the metric describes laboratory activity alone. Supporting subgroup strata were defined a priori by bacterial species (the three predominant organisms analysed individually, with the two *Escherichia* species retained only in overall counts owing to small numbers) and by specimen type. Colistin-based interpretation additionally underpinned two derived, descriptive in vitro metrics: the incremental coverage attributable to cefiderocol over colistin alone, and the incremental diagnostic yield, expressed as the number of isolates that would need to undergo paired testing to identify one additional isolate that is colistin-resistant yet cefiderocol-susceptible (the reciprocal of the absolute proportion of such isolates). The denominator of the incremental diagnostic yield is all matched isolates, that is, every isolate with an interpretable result for both agents, and not the colistin-resistant subgroup alone; the yield, therefore, expresses how many isolates must be submitted for paired testing, not how many colistin-resistant isolates must be found, in order to identify one additional colistin-resistant/cefiderocol-susceptible isolate. Both are diagnostic performance measures of the testing strategy and carry no implication of clinical benefit. Isolates lacking an interpretable colistin result (n = 25) were excluded from paired analyses but retained in single-agent cefiderocol analyses; this differential denominator is reported transparently in each table. The 25 non-interpretable colistin results reflected technical limitations inherent to broth microdilution polymyxin testing rather than any single specimen category: 15 arose from skipped wells or trailing/incomplete growth endpoints that precluded a definitive MIC read, 6 were invalidated by failed growth-control or purity checks on repeat, and 4 corresponded to insufficient residual isolate for confirmatory testing. They were distributed across species broadly in proportion to overall representation (*Klebsiella pneumoniae*, n = 15; *Pseudomonas aeruginosa*, n = 7; *Escherichia coli*, n = 2; *Acinetobacter baumannii*, n = 1) and across specimen types, with no disproportionate clustering in any category.

### 2.4. Statistical Analysis

All analyses were performed in Python v3.11.4 (Python Software Foundation, Wilmington, DE, USA) using the SciPy v1.11.1 and statsmodels v0.14.0 libraries. Continuous variables were summarised as mean ± standard deviation or median with interquartile range according to distributional shape, assessed by the Shapiro–Wilk test. Age departed significantly from normality (W = 0.97, *p* < 0.001) and was consequently compared between cefiderocol phenotypes using the Mann–Whitney U test, with the Welch *t*-test reported for corroboration. Categorical proportions were compared using the Pearson χ^2^ test with continuity correction, with the Fisher exact test substituted where expected cell counts fell below five. Ninety-five percent confidence intervals for proportions were calculated by the Wilson score method, which maintains nominal coverage better than the normal approximation in the small strata present in this dataset.

Paired agreement between cefiderocol and colistin phenotypes was evaluated by the McNemar test, with both the continuity-corrected χ^2^ and the exact binomial variant reported, and chance-corrected agreement was quantified by Cohen’s κ with an asymptotic 95% confidence interval. The relationship between the two agents’ log_2_-transformed MICs was assessed by the Spearman rank correlation coefficient and their paired MIC magnitudes compared by the Wilcoxon signed-rank test. Independent predictors of cefiderocol resistance were identified by multivariable binary logistic regression, incorporating age (per decade), sex, bacterial species (reference category *K. pneumoniae*), and colistin resistance, with results expressed as adjusted odds ratios with 95% confidence intervals and model fit summarised by the McFadden pseudo-R^2^. An ordered trend in cefiderocol resistance across age tertiles was tested by the Cochran–Armitage test for trend. MIC_50_ and MIC_90_ values were derived from the cumulative distribution using the higher-value interpolation convention, and geometric means were computed on the untransformed dilution scale. Two-sided *p*-values below 0.05 were considered statistically significant, and no imputation was performed for missing colistin results. The paired categorical comparison of cefiderocol and colistin susceptibility, performed in the breakpoint-valid subpopulation defined in Section 2.2, was the primary analysis; the regression, trend, and MIC-correlation analyses were pre-specified as secondary and are reported as exploratory. Because EUCAST publishes no clinical cefiderocol breakpoint for *A. baumannii* and no species-specific breakpoint for *E. vulneris*, categorical analyses that include these organisms cannot serve as the primary analysis; the corresponding analyses in the full 236-isolate collection—categorical agreement, McNemar testing, Cohen’s κ, conditional in vitro susceptibility, incremental in vitro coverage and incremental diagnostic yield—are, therefore, reported as secondary descriptive analyses, and the *A. baumannii* cefiderocol data are additionally presented as an MIC distribution set against the three EUCAST evidence tiers. Because de-duplication was applied per species rather than per patient, two further sensitivity analyses were pre-specified: the primary paired analysis was repeated after restricting the dataset to the first isolate obtained from each patient, and the primary logistic regression model was refitted with cluster-robust (Huber–White) standard errors clustered on patient identifier. For species lacking a species-specific cefiderocol breakpoint, MIC values are summarised as full distributions with MIC_50_, MIC_90_ and cumulative percentages rather than as susceptible or resistant proportions.

## 3. Results

Baseline characteristics are summarised in Table 4. Of the 236 XDR isolates, 194 (82.2%) were cefiderocol-non-resistant and 42 (17.8%) were cefiderocol-resistant; 8 isolates, all *K. pneumoniae* with a cefiderocol MIC of exactly 4 mg/L, fell in the susceptible-at-increased-exposure (I) category and are counted with the susceptible isolates in this dichotomy, while *A. baumannii* and *E. vulneris* are categorised here only descriptively against the PK/PD value of 2 mg/L. Mean age was 68.0 ± 12.2 years in the non-resistant group versus 62.5 ± 14.8 years in the resistant group (Mann–Whitney U, *p* = 0.041; Welch *t*-test, *p* = 0.023). Sex distribution did not differ (males 63.4% versus 64.3%; Fisher exact, *p* = 1.000). *K. pneumoniae* accounted for 50.0% of non-resistant and 57.1% of resistant isolates, with no significant difference across the five-species table (χ^2^ = 4.52, *p* = 0.341), and specimen source likewise showed no association (χ^2^ = 2.68, *p* = 0.613). Colistin resistance was present in 39.2% of cefiderocol-non-resistant and 47.5% of cefiderocol-resistant isolates; after adjustment for age, sex, and species, this association was not significant (*p* = 0.663).

**Table 4:** Baseline characteristics of the study population, stratified by cefiderocol phenotype (n = 236).

Characteristic	Cefiderocol-S or I (n = 194)	Cefiderocol-R (n = 42)	*p*-Value	Statistical Test
Age, mean ± SD (years)	68.0 ± 12.2	62.5 ± 14.8	0.023	Welch *t*-test
Age, median (IQR)	70 (61–76)	66 (54–73)	0.041	Mann–Whitney U
Male sex, n (%)	123 (63.4)	27 (64.3)	1.000	Fisher exact
Female sex, n (%)	71 (36.6)	15 (35.7)	—	—
*K. pneumoniae*, n (%)	97 (50.0)	24 (57.1)	0.341	χ^2^ (5 species)
*A. baumannii*, n (%)	71 (36.6)	12 (28.6)	—	—
*P. aeruginosa*, n (%)	21 (10.8)	4 (9.5)	—	—
*Escherichia* spp., n (%)	5 (2.6)	2 (4.8)	—	—
Respiratory source, n (%)	100 (51.5)	21 (50.0)	0.613	χ^2^ (specimens)
Colistin-resistant, n (%) *	67 (39.2)	19 (47.5)	0.663 ^†^	Logistic (adjusted)

* Among isolates with an interpretable colistin result (n = 211). ^†^ Adjusted for age, sex, and species. Abbreviations: S, susceptible; R, resistant; SD, standard deviation; IQR, interquartile range. I, susceptible at increased exposure. The cefiderocol-S or I column combines the susceptible and susceptible-at-increased-exposure categories; the eight isolates in the I category were all *K. pneumoniae* with an MIC of 4 mg/L. Cefiderocol categories for *A. baumannii* and *E. vulneris* are descriptive PK/PD-based classifications only, as these species have no clinical cefiderocol breakpoint; the primary analysis excluding them is given in Section 3.1 and Table 5.

**Table 5:** Primary analysis: paired cefiderocol and colistin results in the species–agent combinations with EUCAST v16.1 species-specific clinical breakpoints for both agents (n = 150 isolates, 126 with a paired colistin MIC).

Metric	Value [95% CI]
Isolates included, n	150 (*K. pneumoniae* 121, *E. coli* 4, *P. aeruginosa* 25)
Cefiderocol susceptible (S), n/N (%)	112/150 (74.7) [67.2–81.0]
Cefiderocol susceptible, increased exposure (I), n/N (%)	8/150 (5.3)
Cefiderocol S or I, n/N (%)	120/150 (80.0) [72.9–85.6]
Cefiderocol resistant (R), n/N (%)	30/150 (20.0) [14.4–27.1]
Isolates with a paired colistin MIC, n	126
Cefiderocol S or I, paired subset, n/N (%)	97/126 (77.0) [68.9–83.5]
Colistin susceptible, paired subset, n/N (%)	55/126 (43.7) [35.3–52.4]
Cefiderocol S or I/colistin-S, n	43
Cefiderocol S or I/colistin-R, n	54
Cefiderocol R/colistin-S, n	12
Cefiderocol R/colistin-R, n	17
Observed (raw) agreement	47.6% (60/126)
Cohen’s κ (95% CI)	0.020 (−0.144 to 0.183)
McNemar χ^2^ (continuity-corrected), *p*	25.47, *p* < 0.001 (exact binomial *p* < 0.001)
Colistin-resistant isolates, n	71
Conditional in vitro susceptibility to cefiderocol, n/N (%)	54/71 (76.1) [65.0–84.5]
*K. pneumoniae*	51/68 (75.0) [63.6–83.8]
*P. aeruginosa*	3/3 (100) [43.9–100]
Coverage by colistin alone, n/N (%)	55/126 (43.7) [35.3–52.4]
Coverage by cefiderocol alone, n/N (%)	97/126 (77.0) [68.9–83.5]
Coverage by either agent, n/N (%)	109/126 (86.5) [79.5–91.4]
Incremental in vitro coverage (cefiderocol over colistin)	+33.3 percentage points
Incremental diagnostic yield	1 additional isolate per 2.3 matched isolates tested

The primary analysis population comprises the species–agent combinations for which EUCAST v16.1 (2026) publishes species-specific clinical breakpoints for both cefiderocol and colistin. *A. baumannii* (n = 83), for which EUCAST publishes no clinical cefiderocol breakpoint, and *E. vulneris* (n = 3), for which no species-specific breakpoint exists for either agent, are excluded; their cefiderocol MIC distributions are given in Section 3.4, and the corresponding pooled analyses are reported as secondary descriptive analyses in Section 3.2. Cefiderocol non-resistant denotes the susceptible (S) and susceptible-at-increased-exposure (I) categories combined; the I category exists for Enterobacterales at an MIC of 4 mg/L only and not for *P. aeruginosa*. Colistin was interpreted at ≤2 mg/L for Enterobacterales and at the EUCAST parenthetical breakpoint of ≤4 mg/L for *P. aeruginosa*. Confidence intervals are Wilson score intervals. The incremental diagnostic yield is the reciprocal of the proportion of all matched isolates that were colistin-resistant and cefiderocol-non-resistant, its denominator being every isolate with an interpretable result for both agents. Conditional in vitro susceptibility is a laboratory descriptor and does not denote clinical benefit. MIC, minimum inhibitory concentration; CI, confidence interval.

### 3.1. Primary Analysis: Paired Cefiderocol–Colistin Comparison in Species–Agent Combinations with EUCAST Clinical Breakpoints

The primary analysis is restricted to the 150 isolates belonging to species for which EUCAST v16.1 publishes species-specific clinical breakpoints for both agents (*K. pneumoniae*, n = 121; *E. coli*, n = 4; *P. aeruginosa*, n = 25); results are summarised in Table 5. Cefiderocol was categorised as susceptible in 112 of these isolates (74.7%, 95% CI 67.2–81.0) and as susceptible at increased exposure in a further 8 (5.3%), all of them *K. pneumoniae* with an MIC of exactly 4 mg/L, giving 120 of 150 (80.0%, 95% CI 72.9–85.6) susceptible at standard or increased exposure and 30 (20.0%, 95% CI 14.4–27.1) resistant. Among the 126 isolates with an interpretable paired colistin MIC, cefiderocol was non-resistant in 97 (77.0%, 95% CI 68.9–83.5) and colistin susceptible in 55 (43.7%, 95% CI 35.3–52.4). The joint distribution comprised 43 isolates non-resistant to both agents, 17 resistant to both, 54 cefiderocol-non-resistant but colistin-resistant, and 12 in the reverse direction. Raw observed agreement was 47.6% (60/126) and chance-corrected agreement negligible (Cohen’s κ = 0.020, 95% CI −0.144 to 0.183), while the 54:12 asymmetry of the discordant cells was highly significant (McNemar continuity-corrected χ^2^ = 25.47, *p* < 0.001; exact binomial *p* < 0.001). Among the 71 colistin-resistant isolates, 54 (76.1%, 95% CI 65.0–84.5) remained cefiderocol-non-resistant; by species, this was 51 of 68 for *K. pneumoniae* (75.0%, 95% CI 63.6–83.8) and 3 of 3 for *P. aeruginosa* (100%, 95% CI 43.9–100), and no *E. coli* isolate with a paired result was colistin-resistant. Coverage rose from 43.7% with colistin alone to 77.0% with cefiderocol alone and 86.5% (109/126, 95% CI 79.5–91.4) with either agent, an incremental in vitro coverage of 33.3 percentage points. The incremental diagnostic yield was one additional colistin-resistant, cefiderocol-non-resistant isolate for every 2.3 isolates submitted to paired testing, the denominator of that figure being all 126 matched isolates rather than the colistin-resistant subgroup alone.

Two features of this primary analysis differ materially from the pooled figures reported in Section 3.2 and deserve emphasis. First, applying the EUCAST parenthetical colistin breakpoint of ≤4 mg/L to *P. aeruginosa* reduces the colistin-resistant *P. aeruginosa* subgroup from seven isolates to three, so the observation that every colistin-resistant *P. aeruginosa* isolate retained cefiderocol activity now rests on three isolates with a confidence interval spanning 43.9 to 100%; it carries almost no precision and should not be read as a species-specific estimate. Second, the eight *K. pneumoniae* isolates with a cefiderocol MIC of exactly 4 mg/L fall in the susceptible-at-increased-exposure category rather than among resistant isolates and account for the difference between the susceptible-only proportion of 74.7% and the 80.0% susceptible at standard or increased exposure. Had these isolates instead been counted as resistant, as they were in the originally submitted analysis, the conditional in vitro susceptibility would have fallen from 76.1% to 69.0% (49/71) and the incremental in vitro coverage from 33.3 to 27.8 percentage points; the direction and significance of every finding are unaffected, but the magnitude is not, which is why both figures are reported.

### 3.2. Secondary Descriptive Analyses of the Full 236-Isolate Collection

Species-stratified susceptibility is shown in Table 6. Cefiderocol non-resistance was 80.2% (95% CI 72.2–86.3) in *K. pneumoniae*, comprising 73.6% susceptible and a further 6.6% susceptible at increased exposure, and 84.0% (95% CI 65.3–93.6) in *P. aeruginosa*; for *A. baumannii*, which has no clinical cefiderocol breakpoint, 85.5% (95% CI 76.4–91.5) of isolates were inhibited at the PK/PD value of 2 mg/L, which is a descriptive figure and not a susceptibility category. There was no significant heterogeneity across the five species (χ^2^ = 4.52, df = 4, *p* = 0.341), and the *K. pneumoniae* versus *A. baumannii* contrast did not approach significance (Fisher exact, OR = 0.68, *p* = 0.355). Colistin susceptibility varied more widely: 35.8% (95% CI 27.4–45.3) in *K. pneumoniae*, 81.7% (95% CI 72.0–88.6) in *A. baumannii*, and 83.3% (95% CI 60.8–94.2) in *P. aeruginosa* once the EUCAST parenthetical breakpoint of ≤4 mg/L is applied to that species—a range of 45.9 percentage points between the two dominant species. Overall, 82.2% of isolates were cefiderocol-non-resistant (194/236) and 59.2% colistin-susceptible (125/211). Both *Escherichia* subgroups were fully colistin-susceptible. The pooled overall susceptibility figures combine species with markedly different resistance epidemiology and are presented as an in vitro descriptive summary; the species-stratified estimates should be used for interpretation.

Specimen-stratified susceptibility is shown in Table 7. Cefiderocol non-resistance by specimen was 86.7% for urine (39/45), 86.7% for purulent secretions (26/30), 80.0% for blood (8/10), 81.8% for respiratory specimens (99/121), and 73.3% for catheter isolates (22/30). The χ^2^ test across specimen types was not significant (χ^2^ = 2.68, df = 4, *p* = 0.613). The catheter stratum carried the widest Wilson confidence interval (55.6–85.8%), reflecting its sample of thirty isolates, and overlapped the intervals of all other strata.

Paired phenotype concordance is summarised in Table 8. Among the 211 isolates with both results available, 104 were non-resistant to both agents, 19 were resistant to both, 67 were cefiderocol-non-resistant but colistin-resistant, and 21 were cefiderocol-resistant but colistin-susceptible. Raw observed agreement was 58.3% (123/211), while chance-corrected agreement was negligible (Cohen’s κ = 0.058, 95% CI −0.093 to 0.208). The discordant cells were asymmetric in a ratio of 67:21, and the McNemar test confirmed this imbalance as significant by both the continuity-corrected χ^2^ (23.01, *p* < 0.001) and the exact binomial variant (*p* < 0.001). Because this pooled table assigns cefiderocol categories to *A. baumannii* and *E. vulneris* on a PK/PD rather than a clinical basis, it is a secondary descriptive analysis; the corresponding primary result, restricted to species with clinical breakpoints, is given in Table 5, where agreement is lower still (κ = 0.020) and the discordance wider (54:12).

Species-specific MIC metrics are summarised in Table 9. For *K. pneumoniae*, cefiderocol MIC_50_ and MIC_90_ were 1 and 8 µg/mL (geometric mean 1.39 µg/mL) versus 8 and 16 µg/mL for colistin (geometric mean 4.36 µg/mL), a three-dilution separation at MIC_50_. For *A. baumannii*, the two agents shared an identical MIC_50_ of 1 µg/mL and MIC_90_ of 4 µg/mL, with geometric means of 0.90 and 1.12 µg/mL respectively. For *P. aeruginosa*, cefiderocol MIC_50_ was 0.5 µg/mL with an MIC_90_ of 8 µg/mL. Across all 206 isolates with paired MIC values, colistin MICs were significantly higher than cefiderocol MICs (Wilcoxon signed-rank, W = 3078, *p* < 0.001). The MIC values themselves are unaffected by the interpretive criteria; what the corrected breakpoints change is their categorisation. Eight *K. pneumoniae* isolates sat at exactly 4 mg/L of cefiderocol, the single dilution that constitutes the susceptible-at-increased-exposure category for Enterobacterales, and four *P. aeruginosa* isolates sat at exactly 4 mg/L of colistin, the value that the EUCAST parenthetical breakpoint for that species places in the susceptible category.

**Figure 1:**
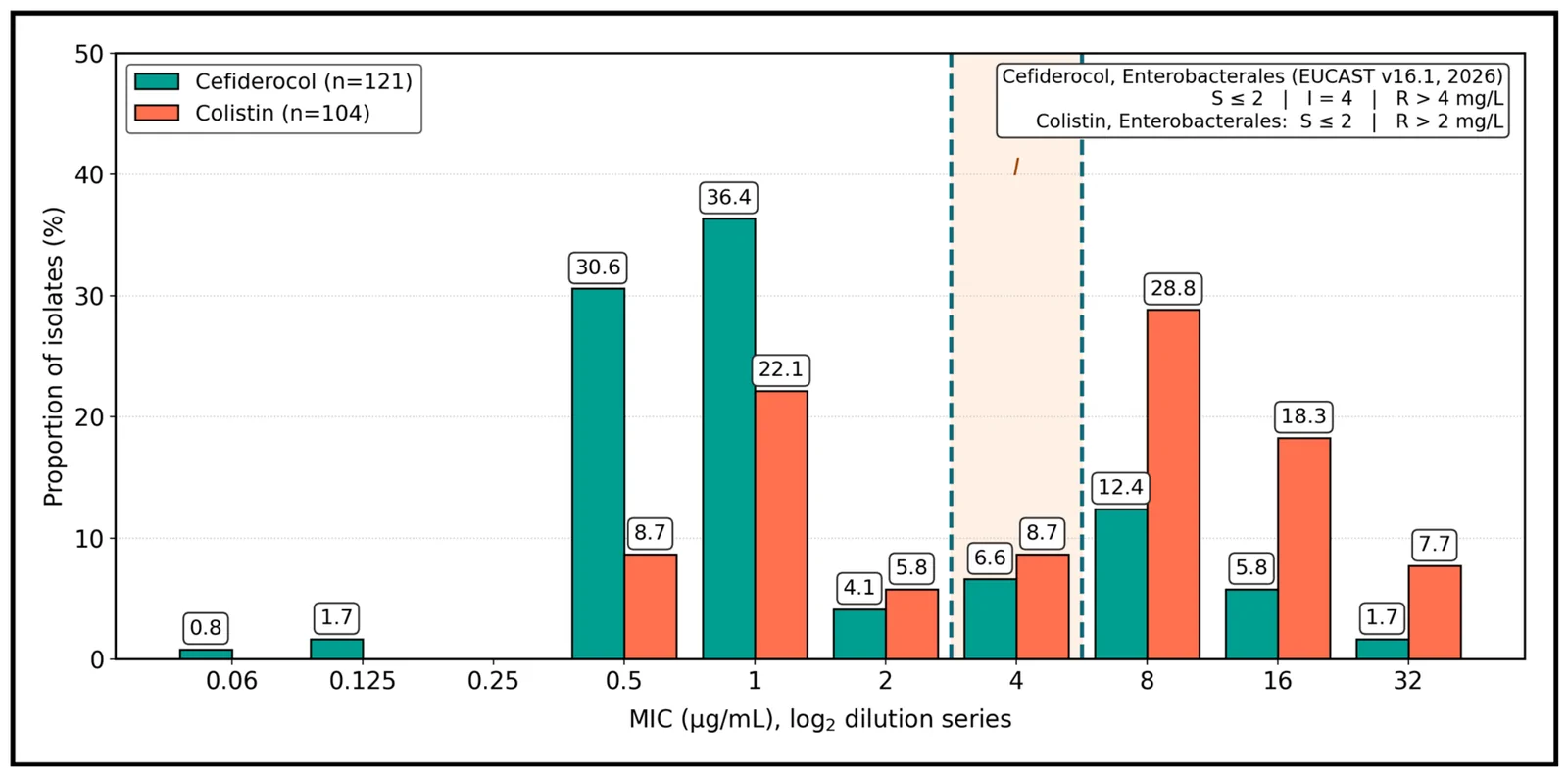
Minimum inhibitory concentration distribution of cefiderocol and colistin against XDR *Klebsiella pneumoniae* (n = 121 cefiderocol, n = 104 colistin). The shaded band at 4 µg/mL marks the susceptible-at-increased-exposure (I) category applied to cefiderocol against Enterobacterales under EUCAST v16.1 (2026); isolates to its left are susceptible (MIC ≤ 2 µg/mL) and those to its right resistant (MIC > 4 µg/mL). For colistin against Enterobacterales, the breakpoint is MIC ≤ 2 µg/mL with no I category. The dashed vertical lines delimit that band. The figure has been redrawn for this revision to show the corrected interpretive categories.

Figure 1 shows that cefiderocol MICs against *K. pneumoniae* clustered at the low end of the dilution series, with 33.1% of isolates at ≤0.5 µg/mL, 30.6% at 0.5 µg/mL, and 36.4% at the modal value of 1 µg/mL; 69.4% fell at ≤1 µg/mL. The colistin distribution was shifted rightward with its mode at 8 µg/mL (28.8%), and 52.9% of isolates (55/104) at or above 8 µg/mL. Only 8.7% of colistin MICs were ≤0.5 µg/mL. The cefiderocol distribution was bimodal, with a susceptible mode at 0.5–1 µg/mL, 4.1% of isolates at the intervening 2 µg/mL dilution, 6.6% (8/121) at exactly 4 µg/mL—the single dilution that EUCAST v16.1 assigns to the susceptible-at-increased-exposure category for Enterobacterales—and a resistant shoulder at 8–32 µg/mL comprising the remaining 19.8%. Cumulative non-resistance at the 4 µg/mL boundary was, therefore, 80.2%, against 73.6% susceptible at the ≤2 µg/mL boundary.

**Figure 2:**
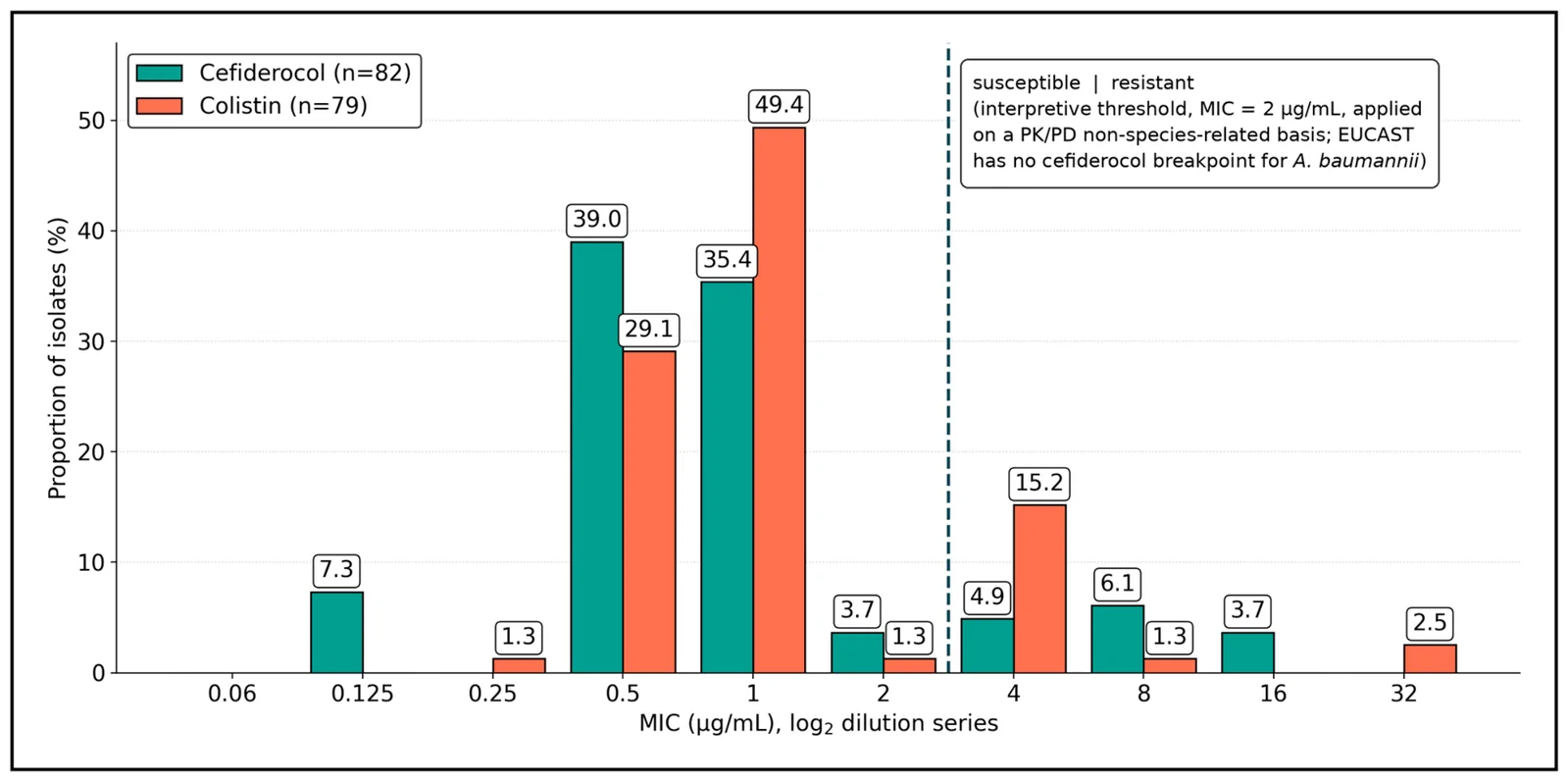
Minimum inhibitory concentration distribution of cefiderocol and colistin against XDR *Acinetobacter baumannii* (n = 82 cefiderocol, n = 79 colistin). The dashed vertical line marks the 2 µg/mL interpretive threshold. EUCAST publishes no clinical cefiderocol breakpoint for *A. baumannii*; it grades MICs of ≤0.5 mg/L, 1–2 mg/L and >2 mg/L by level of evidence instead of defining susceptible and resistant categories. The 2 µg/mL line, therefore, marks the boundary between the upper two evidence tiers and is not a susceptibility breakpoint, and the cefiderocol data for this species must be read as an MIC distribution rather than as a categorical susceptibility result (Section 3.4). For colistin, the value corresponds to the EUCAST species-specific breakpoint for *A. baumannii*.

Figure 2 shows that against *A. baumannii*, the cefiderocol and colistin distributions overlapped closely, both peaking between 0.5 and 1 µg/mL; 74.4% of cefiderocol MICs and 78.5% of colistin MICs fell within this two-dilution window. Cumulative inhibition at the 2 µg/mL interpretive threshold was 85.4% for cefiderocol and 81.0% for colistin, a difference of 4.4 percentage points. Cefiderocol inhibited 7.3% of isolates at 0.125 µg/mL, where colistin inhibited none. At the upper end, 6.1% of cefiderocol MICs reached 8 µg/mL and 3.7% reached 16 µg/mL, versus 1.3% and 0% for colistin; the colistin-resistant fraction was concentrated at 4 µg/mL (15.2%), with 2.5% at 32 µg/mL.

**Figure 3:**
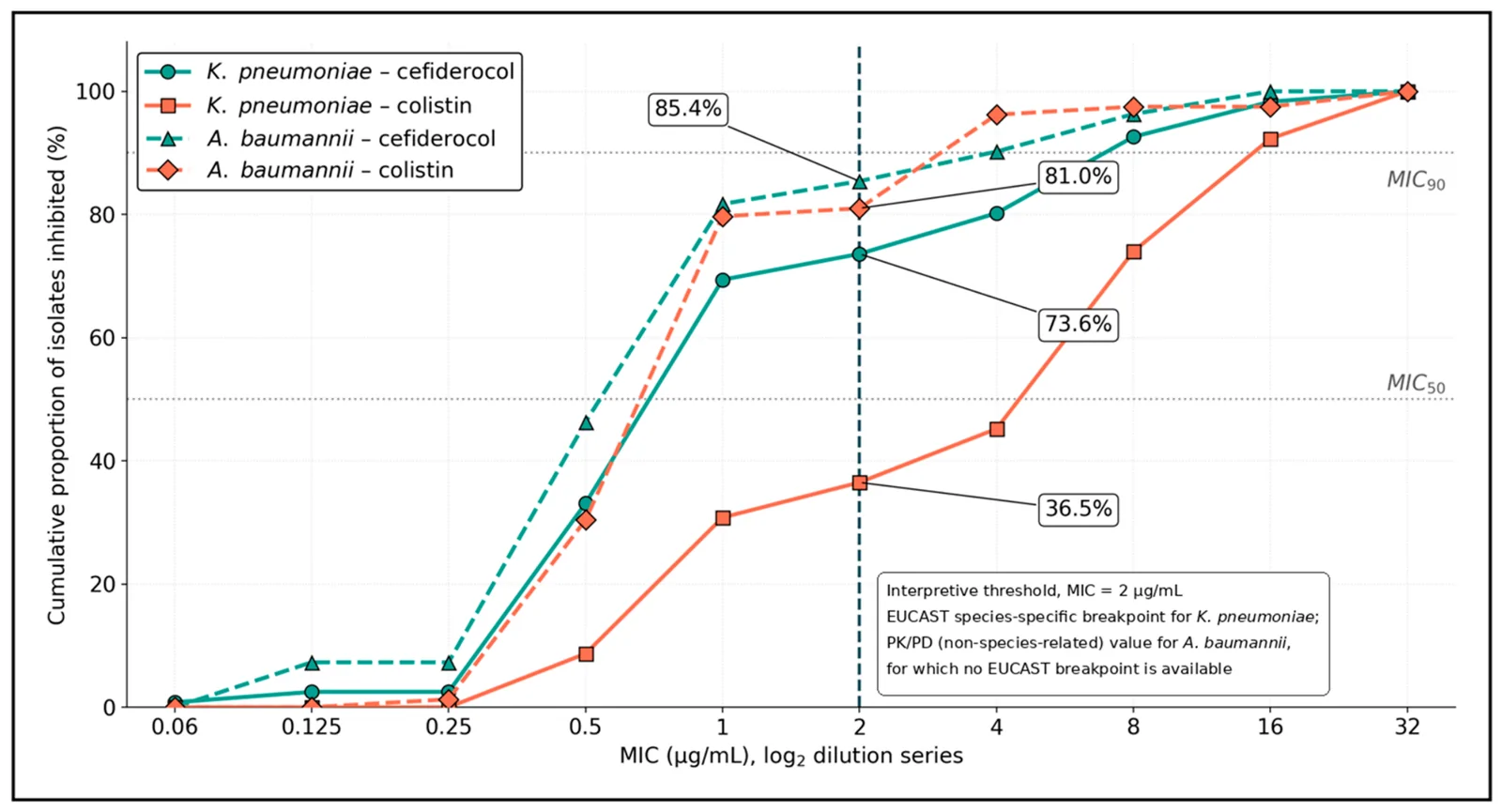
Cumulative MIC inhibition curves for cefiderocol and colistin against *K. pneumoniae* and *A. baumannii*. Horizontal reference lines mark the 50% and 90% inhibition levels; the vertical dashed line marks the 2 µg/mL interpretive threshold, which corresponds to the upper limit of the EUCAST susceptible category for cefiderocol against *K. pneumoniae*—isolates at 4 µg/mL falling in the susceptible-at-increased-exposure category, at which cumulative non-resistance reaches 80.2%—and, for *A. baumannii* cefiderocol, to the boundary between the upper two EUCAST evidence tiers rather than to any susceptibility breakpoint.

Figure 3 presents the cumulative inhibition curves separated by species. Against *A. baumannii*, the cefiderocol and colistin curves tracked closely, both crossing 50% between 0.5 and 1 µg/mL and reaching 85.4% and 81.0%, respectively, at the 2 µg/mL interpretive threshold. Against *K. pneumoniae*, the curves diverged: cefiderocol reached 73.6% cumulative inhibition at the ≤2 µg/mL susceptible boundary and 80.2% at the 4 µg/mL upper limit of the susceptible-at-increased-exposure category, versus 36.5% for colistin at its ≤ 2 µg/mL breakpoint, and colistin did not attain 50% inhibition until 8 µg/mL, four dilutions above the breakpoint. Cefiderocol against *K. pneumoniae* rose 36.3 percentage points across the single 0.5-to-1 µg/mL step and 4.2 points across the next, whereas colistin against *K. pneumoniae* ascended gradually, gaining 28.8 points between 4 and 8 µg/mL.

The conditional in vitro susceptibility of cefiderocol among colistin-resistant isolates is summarised in Table 10. In the primary breakpoint-valid population, 54 of the 71 colistin-resistant isolates (76.1%, 95% CI 65.0–84.5) remained cefiderocol-non-resistant; in the secondary descriptive analysis of the full collection, 67 of 86 (77.9%, 95% CI 68.1–85.4) did so. By species, conditional in vitro susceptibility was 75.0% for *K. pneumoniae* (51/68, 95% CI 63.6–83.8) and 100% for *P. aeruginosa* (3/3, 95% CI 43.9–100), while for *A. baumannii,* 13 of 15 colistin-resistant isolates (86.7%, 95% CI 62.1–96.3) had a cefiderocol MIC at or below the PK/PD value of 2 mg/L—a descriptive count that carries no categorical interpretation, since this species has no clinical cefiderocol breakpoint. *K. pneumoniae* contributed 68 of the 86 colistin-resistant isolates and, therefore, dominates every pooled estimate. All point estimates lay above 50%, a level shown only as a visual reference rather than a pre-specified hypothesis threshold. The *P. aeruginosa* estimate now rests on three colistin-resistant isolates rather than seven, because the EUCAST parenthetical breakpoint of ≤4 mg/L reclassifies four of the seven previously counted as resistant; its confidence interval spans 43.9 to 100% and it must not be read as a reliable species-specific estimate. Neither Escherichia subgroup contributed a colistin-resistant isolate with a paired result.

**Figure 4:**
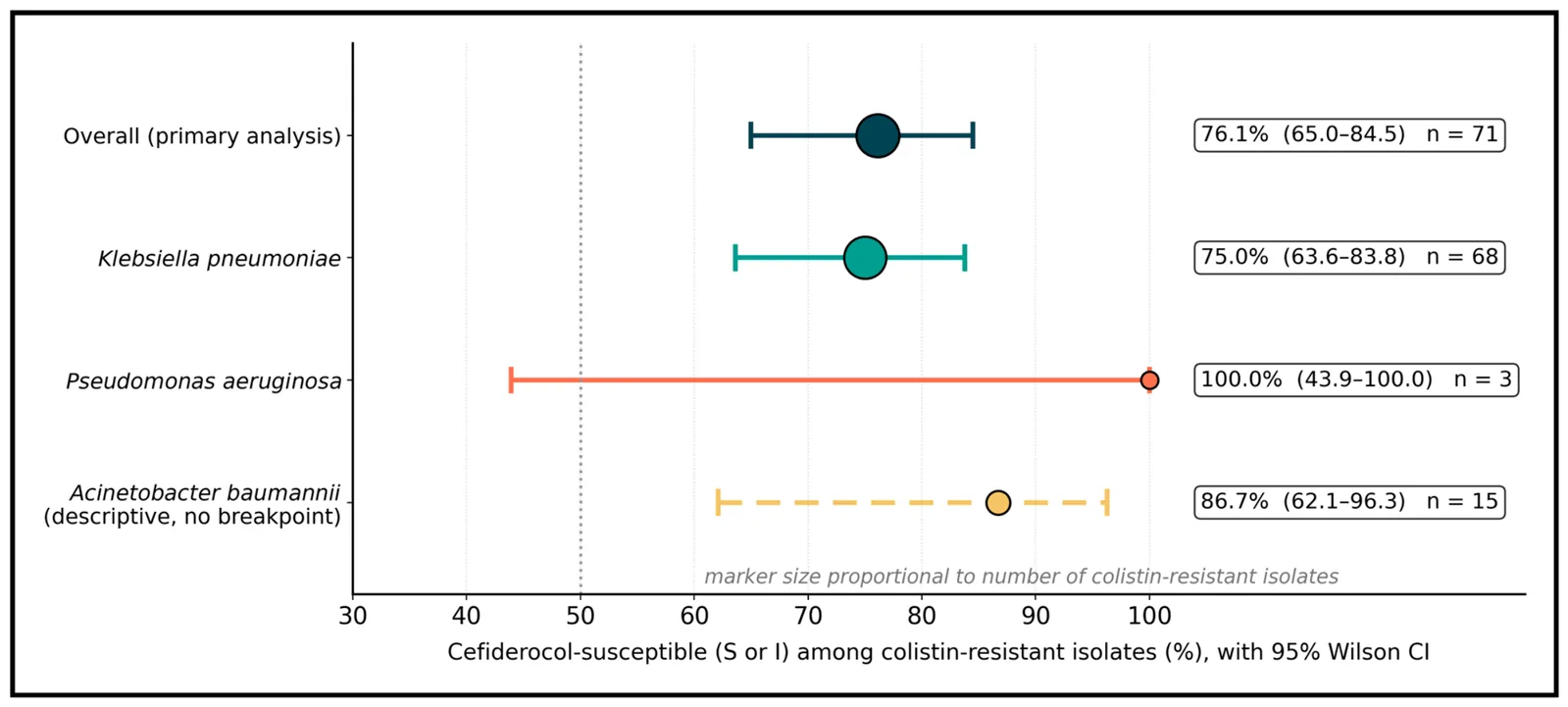
Forest plot of the conditional in vitro susceptibility of cefiderocol—the proportion of colistin-resistant isolates remaining cefiderocol-susceptible in vitro—overall and by species, with 95% Wilson confidence intervals. Marker size is proportional to the number of colistin-resistant isolates in each subgroup. The overall estimate is that of the primary breakpoint-valid population (*K. pneumoniae*, *E. coli*, *P. aeruginosa*); *A. baumannii* is plotted separately with a dashed interval because EUCAST publishes no clinical cefiderocol breakpoint for that species and its value is a descriptive count of isolates with an MIC ≤ 2 mg/L. Colistin resistance in *P. aeruginosa* is defined by the EUCAST parenthetical breakpoint of >4 mg/L. The figure has been redrawn for this revision.

Figure 4 summarises the pattern of conditional in vitro susceptibility: the conditional in vitro susceptibility of cefiderocol given colistin resistance was 76.1% (95% CI 65.0–84.5) across the 71 colistin-resistant isolates of the primary breakpoint-valid population. Subgroup estimates were 75.0% for *K. pneumoniae* (95% CI 63.6–83.8, n = 68) and 100% for *P. aeruginosa* (95% CI 43.9–100, n = 3), with *A. baumannii* shown separately and with a dashed interval, at 86.7% (95% CI 62.1–96.3, n = 15), because that value is a descriptive count against the PK/PD threshold rather than a susceptibility category. The 50% level is shown only as a visual reference and was not a pre-specified hypothesis threshold. Confidence interval width varied inversely with subgroup size, spanning 20.2 percentage points for *K. pneumoniae* and 56.1 points for *P. aeruginosa*, whose interval is now so wide that it excludes no clinically meaningful value.

### 3.3. Extended and Exploratory Analyses

Independent predictors of cefiderocol resistance are summarised in Table 11. Two models were fitted. In the primary model, restricted to the breakpoint-valid paired population (n = 126, 29 events), no predictor reached conventional significance apart from age, and even that only marginally: each 10-year increase in age was associated with lower odds of cefiderocol resistance (adjusted OR 0.71, 95% CI 0.52–0.97, *p* = 0.032), while female sex (adjusted OR 0.94, *p* = 0.888), *P. aeruginosa* (adjusted OR 0.79, *p* = 0.740), *E. coli* (adjusted OR 6.42, *p* = 0.142) and colistin resistance (adjusted OR 1.24, 95% CI 0.51–3.02, *p* = 0.639) were not. Model McFadden pseudo-R^2^ was 0.061. In the secondary model fitted to the full paired collection (n = 211, 40 events), age behaved similarly (adjusted OR 0.68, 95% CI 0.51–0.90, *p* = 0.007), and colistin resistance again showed no association (adjusted OR 1.19, 95% CI 0.54–2.61, *p* = 0.663); notably, the lower odds of cefiderocol resistance previously reported for *A. baumannii* no longer reach significance once the intermediate category is applied to Enterobacterales (adjusted OR 0.52, 95% CI 0.22–1.24, *p* = 0.140), because the corrected *K. pneumoniae* reference rate falls from 26.4% to 19.8%. Both models, therefore, account for only a small fraction of the variation in cefiderocol resistance, no candidate confounder reaches significance in the primary model other than age, and the ordered age trend reported below is not statistically significant. The age association is accordingly presented as an exploratory, hypothesis-generating observation and is not advanced as a principal finding of this study. Refitting the primary model with cluster-robust standard errors clustered on patient left the estimates essentially unchanged (age adjusted OR 0.71, 95% CI 0.51–0.99; colistin resistance adjusted OR 1.24, 95% CI 0.50–3.09).

**Figure 5:**
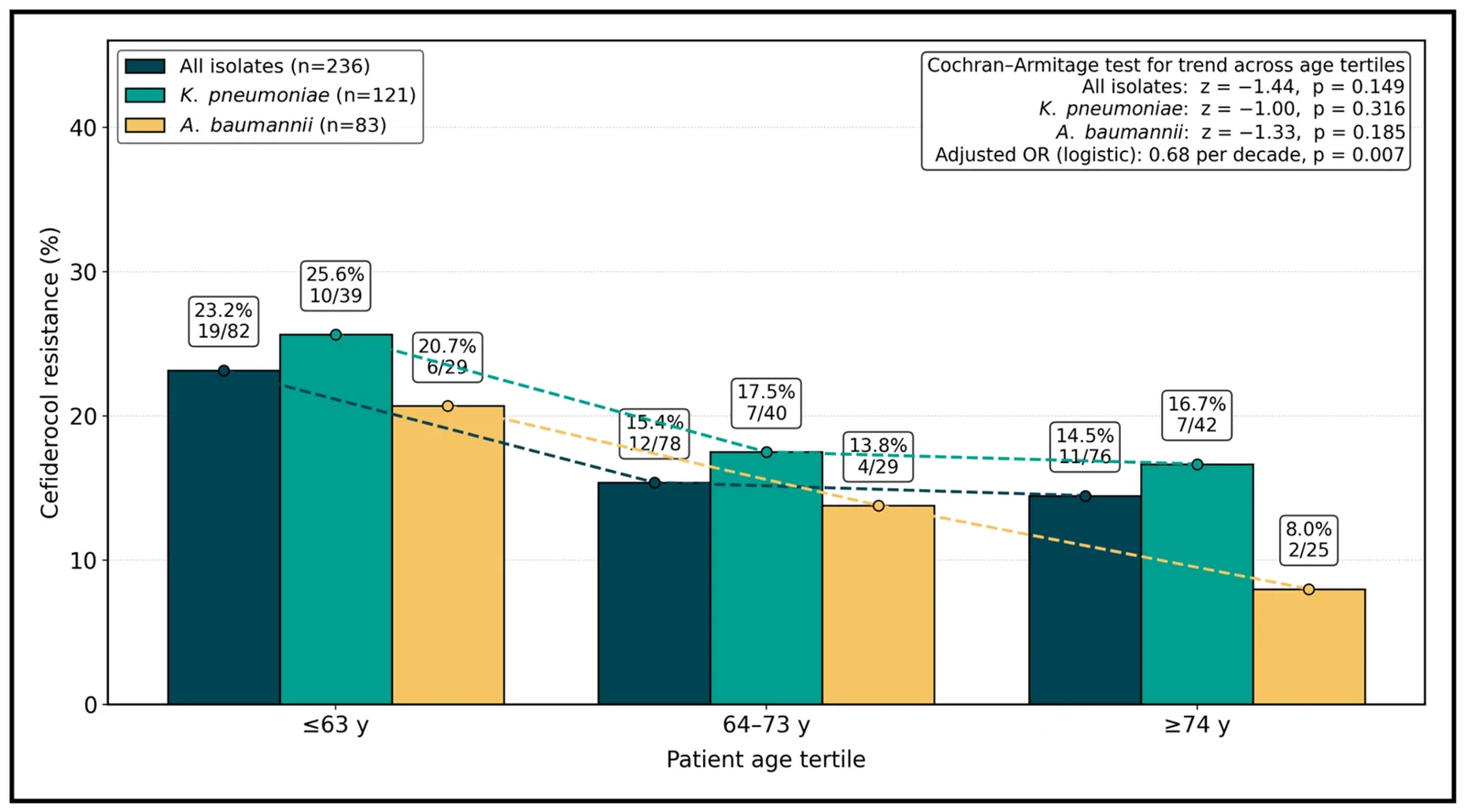
Cefiderocol resistance across patient age tertiles, overall and stratified by species, with 95% Wilson confidence intervals on the overall estimates and Cochran–Armitage trend statistics. The dashed lines connect the resistance estimates across consecutive tertiles to show the direction of the gradient and are not fitted regression lines. Cefiderocol resistance is defined by the corrected species-specific EUCAST v16.1 breakpoints, with Enterobacterales isolates at 4 mg/L classified as susceptible at increased exposure rather than resistant; the figure has been redrawn accordingly. The ordered trend is not statistically significant overall or in either major species, and the panel is presented as exploratory.

Figure 5 shows that cefiderocol resistance declined monotonically across age tertiles, from 23.2% (19/82; 95% CI 15.3–33.5) in patients aged ≤63 years to 15.4% (12/78) at 64–73 years to 14.5% (11/76; 95% CI 8.3–24.1) at ≥74 years—an absolute decrement of 8.7 percentage points (Cochran–Armitage z = −1.44, *p* = 0.149). The same monotonic decline appeared within *K. pneumoniae* (25.6% → 17.5% → 16.7%; z = −1.00, *p* = 0.316) and within *A. baumannii* (20.7% → 13.8% → 8.0%; z = −1.33, *p* = 0.185). The confidence intervals of the two upper tertiles overlapped substantially. The corresponding adjusted odds ratio was 0.68 per decade in the secondary model (*p* = 0.007) and 0.71 per decade in the primary model (*p* = 0.032). Because the ordered trend is non-significant overall and within each major species, while the regression coefficient is significant, this gradient is reported as an exploratory observation and not as a result of this study; the discrepancy between the two tests is itself a reason for caution.

MIC-level concordance between cefiderocol and colistin is summarised in Table 12. Across 206 paired MIC values, the Spearman rank correlation between log_2_-transformed cefiderocol and colistin MICs was weak but statistically significant (ρ = 0.196, *p* = 0.005), corresponding to under 4% shared variance. Median MIC was 1.0 µg/mL for both agents. The mean log_2_ difference was +0.90, indicating colistin MICs approximately 1.87-fold higher than cefiderocol MICs for the same isolate, and the Wilcoxon signed-rank test confirmed this systematic shift (*p* < 0.001). Categorical agreement was negligible in both populations: Cohen’s κ was 0.020 in the primary breakpoint-valid analysis (Table 5) and 0.058 in the secondary analysis of the full collection (Table 8). Because the MIC values themselves are unchanged by the interpretive corrections, the rank correlation, the median MICs and the Wilcoxon result are identical to those originally reported.

Incremental antimicrobial coverage is summarised in Table 13. In the primary breakpoint-valid population, coverage among the 126 paired isolates was 43.7% (55/126) for colistin alone, 77.0% (97/126) for cefiderocol alone, and 86.5% (109/126) for either agent, an absolute incremental coverage of 33.3 percentage points for cefiderocol over colistin. In the secondary analysis of the full collection, the corresponding figures among 211 paired isolates were 59.2% (125/211), 81.0% (171/211) and 91.0% (192/211), an incremental coverage of 21.8 percentage points. The incremental diagnostic yield, the reciprocal of the proportion of all matched isolates that were both colistin-resistant and cefiderocol-non-resistant, corresponded to 1 additional such isolate identified for every 2.3 isolates submitted to paired testing in the primary population (54/126 = 42.9%) and 1 for every 3.1 in the full collection (67/211 = 31.8%); in both cases, the denominator is every isolate with an interpretable result for both agents, not the colistin-resistant subgroup. These coverage figures describe in vitro activity only: they assume that categorical susceptibility equates to a usable therapeutic option and do not account for infection site, pharmacokinetics, toxicity, dosing, undetected resistance mechanisms, or the possibility that some isolates (for example, from catheter tips) represented colonisation rather than infection. Net species-specific gains, after subtracting reverse-discordant cases, were +41.5% for *K. pneumoniae* (gain 48.1%, reverse 6.6%), +4.9% for *A. baumannii* (gain 15.9%, reverse 11.0%, descriptive only) and 0.0% for *P. aeruginosa* (gain 16.7%, reverse 16.7%). The *P. aeruginosa* result is the most consequential change produced by the corrected colistin breakpoint: with colistin resistance defined at >4 mg/L, the three isolates that cefiderocol adds are exactly balanced by the three that colistin covers and cefiderocol does not, so paired testing produces no net in vitro gain in this species, and the case for testing both agents in *P. aeruginosa* rests on the discordance itself rather than on any net increase in coverage.

**Figure 6:**
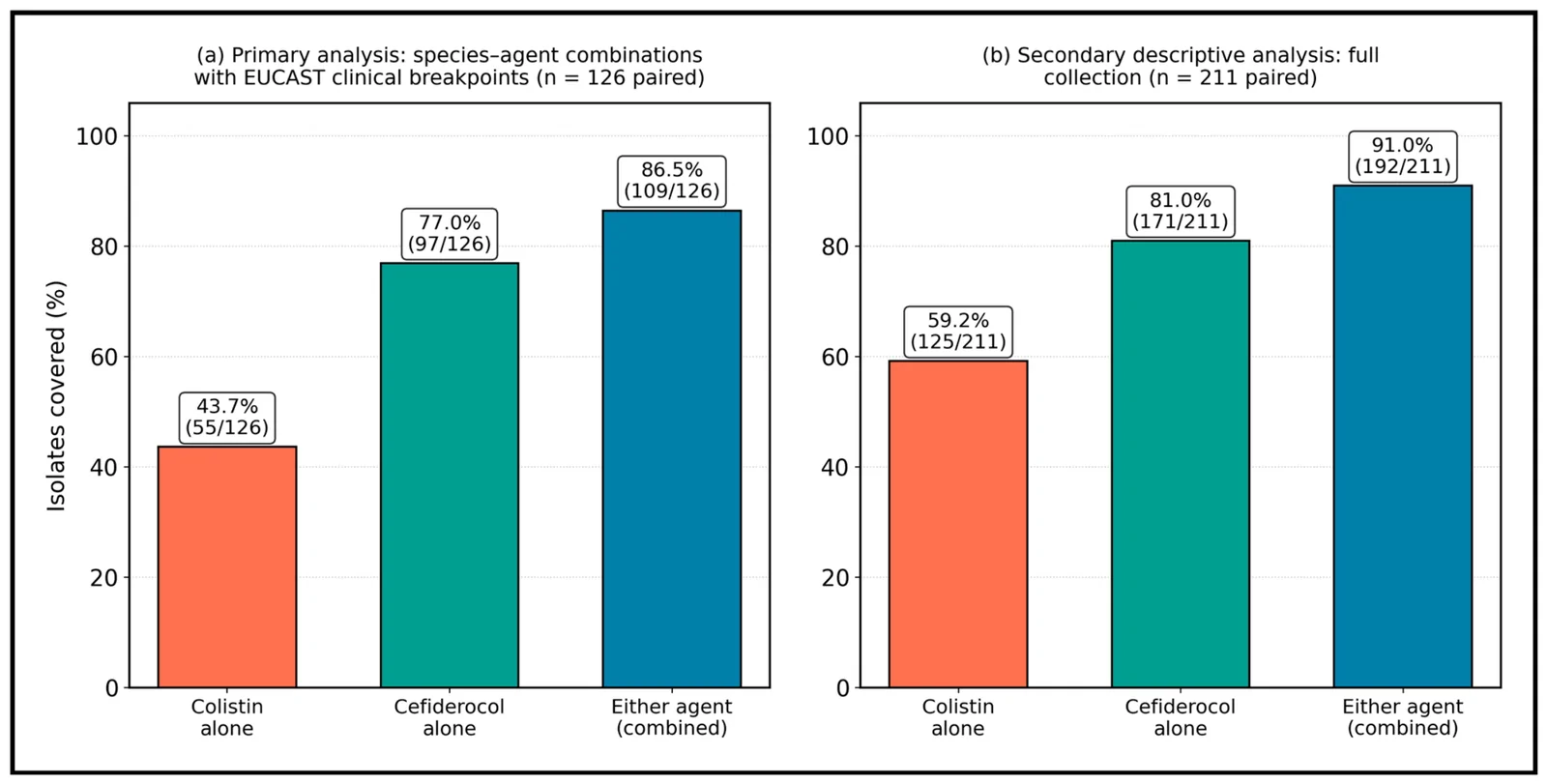
Incremental coverage cascade: proportion of isolates covered by colistin alone, by cefiderocol alone, and by either agent selected according to the antibiogram, shown for (**a**) the primary breakpoint-valid population (n = 126 paired isolates) and (**b**) the full collection as a secondary descriptive analysis (n = 211 paired isolates). Coverage denotes categorical non-resistance in vitro under the species-specific EUCAST v16.1 breakpoints of Table 2 and carries no implication of clinical benefit. The figure has been redrawn for this revision.

Figure 6 illustrates the incremental coverage pattern. In the primary breakpoint-valid population, coverage among the 126 paired isolates rose from 43.7% (55/126) with colistin alone to 77.0% (97/126) with cefiderocol alone, an absolute difference of 33.3 percentage points, and reached 86.5% (109/126) with either agent; the increment from cefiderocol alone to either-agent coverage was 9.5 percentage points, corresponding to the 12 colistin-susceptible but cefiderocol-resistant isolates, and a further 13.5% (17/126) were resistant to both agents and appear in none of the bars. In the secondary analysis of the full collection, the corresponding values were 59.2% (125/211), 81.0% (171/211) and 91.0% (192/211), with 9.0% (19/211) resistant to both agents.

### 3.4. Species Without Clinical Breakpoints, Comparison of the Two Analysis Populations, and Patient-Level Sensitivity Analysis

Because EUCAST publishes no clinical cefiderocol breakpoint for *A. baumannii* and no species-specific breakpoint for *E. vulneris*, cefiderocol results for these organisms are presented as MIC distributions in Table 14 rather than as categorical susceptible or resistant proportions. For *A. baumannii*, EUCAST does not recommend a susceptible-or-resistant classification at all; it instead grades the MIC into three bands supported by different levels of evidence, and the distribution is reported against those bands. The *A. baumannii* distribution (n = 82) was unimodal, with a modal MIC of 0.5 µg/mL (39.0%), MIC_50_ of 1 µg/mL and MIC_90_ of 4 µg/mL; 46.3% of isolates were inhibited at ≤0.5 µg/mL and 85.4% at ≤2 µg/mL, with a right-hand tail of 12 isolates (14.6%) distributed between 4 and 16 µg/mL and no isolate above 16 µg/mL. The three *E. vulneris* isolates had cefiderocol MICs of 0.25, 0.5 and 1 µg/mL. Set against the three EUCAST evidence tiers, 38 of the 82 *A. baumannii* isolates (46.3%) had an MIC of ≤0.5 mg/L, the band for which EUCAST regards the evidence as sufficient; 32 (39.0%) fell in the 1–2 mg/L band, for which the evidence is regarded as insufficient and the response uncertain; and 12 (14.6%) exceeded 2 mg/L, where a response is considered unlikely. Two of the three *E. vulneris* isolates fell in the lowest band and one in the 1–2 mg/L band. No categorical assignment is made for either organism.

Table 15 sets the primary analysis of Section 3.1 alongside the corresponding values from the secondary descriptive analysis of the full collection, so that the consequences of restricting the comparison to species–agent combinations with clinical breakpoints can be read directly. Every primary finding runs in the same direction in both populations, but the magnitudes are not identical, and the two sets of results are, therefore, described as directionally consistent rather than unchanged. Chance-corrected agreement is negligible in both (Cohen’s κ 0.020 versus 0.058), and the discordance is significantly asymmetric in both (McNemar continuity-corrected χ^2^ 25.47 versus 23.01, both *p* < 0.001; exact binomial *p* < 0.001 in both). Conditional in vitro susceptibility is 76.1% (54/71) in the primary population and 77.9% (67/86) in the full collection. Incremental in vitro coverage differs appreciably, however: 33.3 percentage points in the primary population against 21.8 in the full collection, a span of 11.5 percentage points that reflects the much lower colistin susceptibility of the breakpoint-valid species (43.7% versus 59.2% among paired isolates). Excluding *A. baumannii*—the species in which the two agents were most concordant, and the one for which no clinical cefiderocol breakpoint exists—therefore, widens rather than narrows the observed discordance, and the estimate of what paired testing adds should be quoted as a range of 21.8 to 33.3 percentage points depending on the population considered, with the primary breakpoint-valid figure of 33.3 percentage points as the estimate on which clinical–microbiological inference should rest.

Because de-duplication was applied per species rather than per patient, the primary paired analysis was repeated after restricting the dataset to the first isolate obtained from each of the 220 unique patients; the comparison is given in Table 16. This removed 16 isolates (*K. pneumoniae*, n = 8; *A. baumannii*, n = 5; *P. aeruginosa*, n = 3) and left 139 breakpoint-valid isolates, 117 of them with an interpretable paired colistin MIC. Within this patient-level dataset, cefiderocol was non-resistant in 90 of 117 isolates (76.9%, 95% CI 68.5–83.6) and colistin susceptible in 52 (44.4%, 95% CI 35.8–53.5); chance-corrected agreement remained negligible (Cohen’s κ = 0.032, 95% CI −0.139 to 0.203) and the discordance significantly asymmetric (49 versus 11; McNemar continuity-corrected χ^2^ = 22.82, *p* < 0.001). Conditional in vitro susceptibility among the 65 colistin-resistant isolates was 75.4% (49/65, 95% CI 63.7–84.2), incremental in vitro coverage was 32.5 percentage points, and the incremental diagnostic yield 1 additional isolate per 2.4 matched isolates tested. No estimate differs materially from its counterpart in the primary analysis. Refitting the primary logistic regression model with cluster-robust standard errors clustered on patient identifier likewise left the coefficients essentially unchanged (age adjusted OR 0.71, 95% CI 0.51–0.99 against 0.52–0.97 with model-based standard errors; colistin resistance adjusted OR 1.24, 95% CI 0.50–3.09 against 0.51–3.02). The violation of the independence assumption introduced by species-level de-duplication is, therefore, not a material threat to the conclusions of this study.

## 4. Discussion

This within-isolate analysis of XDR Gram-negative bacilli shows that cefiderocol had higher aggregate in vitro activity than colistin, both in the primary breakpoint-valid population (77.0% versus 43.7% among the 126 paired isolates) and in the secondary descriptive analysis of all 236 isolates (82.2% versus 59.2%); because the pooled figure combines species with very different resistance epidemiology, and because it assigns cefiderocol categories to *A. baumannii* for which no clinical breakpoint exists, it should be read as an in vitro descriptive comparison rather than as evidence that cefiderocol is a better therapeutic option, and its more consequential contribution lies in reframing the comparison from parallel antibiograms to a paired discordance model. The near-zero Cohen’s κ—0.020 in the primary analysis and 0.058 in the full collection—and the highly asymmetric McNemar result—54 cefiderocol-non-resistant/colistin-resistant isolates versus 12 in the reverse direction in the primary population, and 67 versus 21 in the full collection—demonstrate that the two agents are not interchangeable measures of a shared underlying resistance state but rather represent discordant susceptibility phenotypes. Neither the McNemar test nor Cohen’s kappa is a test of biological independence: McNemar evaluates the asymmetry of the discordant cells and kappa quantifies agreement beyond chance, so these statistics describe phenotypic discordance and do not establish that the underlying resistance mechanisms are independent. The absence of a statistically significant phenotypic association (adjusted odds ratio for colistin resistance 1.24, 95% CI 0.51–3.02) is consistent with, but does not by itself prove, the distinct pharmacology of cefiderocol’s siderophore-mediated periplasmic entry and its stability to many enzymes across the Ambler classes, neither of which shares any mechanistic overlap with the polymyxin lipopolysaccharide target [13,15].

The principal within-isolate finding is the conditional in vitro susceptibility of cefiderocol given colistin resistance: 76.1% of the colistin-resistant isolates in the primary breakpoint-valid population remained cefiderocol-non-resistant, holding at 75.0% in *K. pneumoniae*—the organism in which colistin has become least reliable, with only 35.8% susceptibility in this cohort. The 100% figure previously reported for *P. aeruginosa* survives the correction only in the trivial sense that all three isolates that remain colistin-resistant under the EUCAST parenthetical breakpoint of ≤4 mg/L are cefiderocol-susceptible; with a confidence interval of 43.9 to 100%, it supports no species-specific claim, and the net in vitro gain from paired testing in *P. aeruginosa* is in fact zero. The derived in vitro metrics are descriptive and do not account for infection site, pharmacokinetics, toxicity, or dosing: an incremental in vitro coverage of 33.3 percentage points in the primary population and 21.8 in the full collection, an incremental diagnostic yield of 1 additional cefiderocol-non-resistant isolate per 2.3 and per 3.1 matched isolates, respectively—the denominator in each case being every isolate tested in parallel, not the colistin-resistant subgroup—and a net species-specific gain peaking at 41.5% for *K. pneumoniae*. Our estimate of conditional in vitro susceptibility is directionally consistent with the small series that first reported an absence of cefiderocol–colistin cross-resistance [25] but is derived from 71 rather than 6 colistin-resistant isolates and, therefore, carries far greater precision. It is also more conservative, and this divergence is informative: our single-centre cefiderocol resistance of 20.0% in the breakpoint-valid population (17.8% across the full collection) sits well above the pooled global estimate but within the range that the meta-analysis of Karakonstantis and colleagues attributed to carbapenem-resistant phenotypes in individual institutions [24] and well above the >96% susceptibility reported by multinational surveillance in unselected populations [18].

The unexpected inverse association between patient age and cefiderocol resistance (adjusted OR 0.71 per decade, *p* = 0.032, in the primary model; 0.68 per decade, *p* = 0.007, in the secondary model) should be regarded as an exploratory, hypothesis-generating observation only and is reported for completeness rather than as one of this study’s principal results. In the multivariable model, older age was associated with lower (not higher) cefiderocol resistance, whereas the Cochran–Armitage age-tertile trend was not statistically significant overall or within either major species; this discrepancy, together with the low model explanatory power (McFadden pseudo-R^2^ = 0.061) and the absence of key covariates such as prior antimicrobial exposure, ward or ICU admission, comorbidities, and prior colonisation, means the association cannot be interpreted causally. Because no clinical, comorbidity, or prior-exposure data were available, this signal cannot be mechanistically attributed and may reflect residual confounding by unmeasured factors such as differential species distribution, healthcare-exposure patterns, ward casemix, or referral pathways across age strata. Notably, the Cochran–Armitage trend across age tertiles was not significant overall (z = −1.44, *p* = 0.149) and was likewise non-significant within both *K. pneumoniae* (*p* = 0.316) and *A. baumannii* (*p* = 0.185), even though the gradient ran in the same direction in each species. The contrast between a significant adjusted regression coefficient and non-significant ordered trends may reflect a modest monotonic effect that the trend test is underpowered to detect, but it is equally consistent with chance, and for that reason, the age finding is deliberately excluded from the summary of principal results in the Abstract and Conclusions. Rather than a biological claim, this observation is best read as a hypothesis-generating pointer for future work coupling microbiological phenotyping with granular patient-level covariates.

The 20.0% cefiderocol resistance observed here in the breakpoint-valid population is high relative to multinational surveillance and warrants mechanistic consideration, even though no molecular characterisation was performed. The mechanisms outlined below are provided as biological background rather than as findings of the present study, which included no molecular characterisation and, therefore, cannot attribute the observed resistance to any specific determinant. Contemporary work has identified several routes to cefiderocol resistance that would be entirely invisible to the phenotypic methods used in this study. Amplification of β-lactamase genes—for example, tandem duplication of *blaSHV-12* within IS26-flanked plasmid segments—can generate dynamic, within-host heteroresistance that is inducible by drug exposure and reversible upon its withdrawal, producing isolates whose apparent MIC depends on recent selection history [30]. Mutations disrupting the TonB-dependent iron-transport apparatus represent a second route, and in metallo-β-lactamase-producing Enterobacterales, such mutations have been shown to arise readily under cefiderocol pressure, sometimes accompanied by small-colony-variant morphologies with implications for both detection and bacterial clearance [31]. A third contributor is active efflux: recent analysis of 370 carbapenem-resistant isolates found that chemical efflux-pump inhibition markedly reduced cefiderocol MICs in resistant strains, alongside β-lactamase variants and altered expression of iron-transporter genes [32]. The bimodal MIC distribution evident in our *K. pneumoniae* population—a dominant susceptible mode at 0.5–1 µg/mL and a distinct resistant shoulder at 8–32 µg/mL, with only 4.1% of isolates at the intervening 2 µg/mL dilution and a further 6.6% at the 4 µg/mL dilution that constitutes the susceptible-at-increased-exposure category—could arise from several phenotypic or technical mechanisms and should be interpreted cautiously; because no molecular testing was performed, this pattern warrants genomic investigation rather than being taken as evidence of discrete genetic resistance determinants.

Translating conditional in vitro susceptibility into clinical benefit requires caution, and three considerations temper any direct extrapolation. First, the mortality imbalance observed in CREDIBLE-CR, concentrated among patients with Acinetobacter infections, has never been fully explained and continues to constrain confident first-line use of cefiderocol in exactly the critically ill population represented in our cohort [17]. Second, real-world experience suggests that susceptibility on a report does not guarantee cure: in a multicentre cohort of 114 immunocompromised patients treated with cefiderocol for multidrug-resistant Gram-negative infection, clinical success at day 28 reached only 53.3% with 37.7% overall mortality, and on-therapy resistance acquisition, though uncommon, was documented [33]. Third, and most directly relevant to a microbiology-based study such as ours, the categorisation of cefiderocol susceptibility is itself methodologically fragile: in a series of carbapenemase-producing isolates from Ukrainian patients, reported susceptibility varied from 30% to 97% for the same organisms depending on whether microcolonies were read, whether EUCAST or CLSI breakpoints were applied, and whether disc diffusion or broth microdilution was used [34]. Our uniform use of iron-depleted broth microdilution, with daily verification of adequate iron depletion through control-strain MICs, together with strictly species-specific EUCAST v16.1 criteria applied only where a clinical breakpoint exists, mitigates but does not eliminate this source of variability, and it means our estimates are not automatically comparable with series using CLSI breakpoints, under which the same isolates would appear more susceptible.

A parallel caveat applies to the colistin arm and, if anything, strengthens the argument for cefiderocol testing. Categorical colistin susceptibility is an imperfect surrogate for in vivo activity because heteroresistant subpopulations—resistant minorities coexisting within an apparently susceptible population—are common in *A. baumannii* and are systematically missed by routine diagnostics, including broth microdilution, requiring population analysis profiling for reliable detection [35]. Distinct heteroresistance phenotypes have been described, some of which cannot be eradicated even at high colistin concentrations while others revert on passage [36], meaning that a proportion of the isolates we classified as colistin-susceptible may in practice fail colistin therapy. Our estimate of the incremental in vitro coverage attributable to cefiderocol is, therefore, likely to be conservative rather than inflated. A second caveat runs in the opposite direction and concerns the colistin breakpoint itself: for *P. aeruginosa*, EUCAST publishes the breakpoint in parentheses precisely because the supporting evidence is limited, and applying it at ≤4 mg/L rather than at the ≤2 mg/L value used for the other species more than halves the colistin-resistant *P. aeruginosa* subgroup and abolishes the net in vitro gain from paired testing in that species. Any statement about what cefiderocol adds in *P. aeruginosa* is, therefore, contingent on a breakpoint that EUCAST itself qualifies. It should nonetheless be emphasised that cefiderocol is not the only alternative to a failing polymyxin: ceftazidime–avibactam has demonstrated a mortality advantage over colistin-based regimens in carbapenem-resistant Enterobacterales bacteraemia in meta-analysis [37], and current guidance reserves distinct roles for sulbactam–durlobactam, ceftazidime–avibactam with aztreonam, and cefiderocol according to organism and resistance mechanism [38]. Indeed, 27 isolates in our cohort retained susceptibility to aztreonam/avibactam or to a ceftazidime–avibactam–aztreonam combination, and site-specific considerations—notably the favourable urinary pharmacology exploited in the cefiderocol registration programme [39]—further modulate agent selection. The correct inference from our data is, therefore, not that cefiderocol should replace colistin but that the two agents fail independently, so testing both materially expands the therapeutic option set.

## 5. Study Limitations

Several limitations temper the interpretation of these findings. First, this was a retrospective, single-centre laboratory study without access to patient-level clinical data; comorbidities, prior antimicrobial exposure, infection versus colonisation status, and, critically, clinical outcomes were unavailable, so the analysis is confined to microbiological endpoints and cannot demonstrate that in vitro cefiderocol susceptibility translates into improved survival or cure. Second, the sample was unevenly distributed across species and specimens, with small subgroups (*P. aeruginosa*, both *Escherichia* species, and blood cultures) yielding wide confidence intervals and limiting the precision of stratified estimates; the 100% *P. aeruginosa* conditional in vitro susceptibility, in particular, now rests on only three isolates once the EUCAST parenthetical colistin breakpoint of ≤4 mg/L is applied to that species. Third, 25 isolates lacked an interpretable colistin result and were necessarily excluded from paired analyses, introducing potential selection bias if missingness was non-random. Fourth, categorical assignment near the breakpoint remains sensitive to the interpretive framework. This revision applies the species-specific EUCAST v16.1 (2026) criteria in full, including the intermediate (susceptible, increased exposure) category for cefiderocol against Enterobacterales at 4 mg/L and the parenthetical colistin breakpoint of ≤4 mg/L for *P. aeruginosa*, but divergence between EUCAST and CLSI thresholds and the well-documented interlaboratory variability in both cefiderocol iron-depleted broth microdilution and colistin MIC determination may still materially affect assignment near a breakpoint [24,34]. The limitation is addressed by making the breakpoint-valid population the primary analysis (Table 5), by presenting species without a clinical cefiderocol breakpoint as MIC distributions set against the EUCAST evidence tiers (Table 14), and by reporting the pooled comparison only as a secondary descriptive analysis (Table 15). The direction of every finding is preserved across the two populations, but the magnitude is not: incremental in vitro coverage ranges from 21.8 to 33.3 percentage points, and the two sets of results are, therefore, described as directionally consistent rather than unchanged. Categorical cefiderocol assignment for *A. baumannii* in the secondary tables remains PK/PD-based and provisional and should not be used clinically. Fifth, no molecular characterisation of resistance mechanisms was performed—carbapenemase genotype, *mcr* status, *mgrB* alterations, and siderophore-receptor or TonB mutations were all unavailable—so the phenotypic discordance observed could not be attributed to independent resistance mechanisms at the genetic level, and heteroresistant subpopulations in either arm would have gone undetected [30,35]. Sixth, the absence of an independent reference method precludes formal essential- and categorical-agreement error-rate analysis, and no repeat testing was performed to quantify within-laboratory reproducibility. Finally, the surveillance window spans a single institution over eight months, so local clonal expansion could inflate apparent resistance rates in a manner that would not generalise. A further constraint concerns the unit of analysis: de-duplication was applied per species rather than per patient, so 16 of the 236 isolates (6.8%) came from a patient already represented by a different species, and the paired analyses assume an independence that these isolates do not strictly satisfy. A patient-level sensitivity analysis restricted to the first isolate per patient and a cluster-robust refitting of the primary regression left every primary estimate essentially unchanged (Table 16), but residual within-patient correlation cannot be excluded. Related to this, the XDR definition is less discriminating in *A. baumannii* than in the other species, because only four antimicrobial categories retain EUCAST breakpoints for that organism once the two study agents are set aside, so almost any carbapenem-resistant *A. baumannii* isolate satisfies the definition; for this species, the category-level data of Supplementary Tables S1 and S2 are more informative than the XDR label itself. These constraints collectively frame this study as hypothesis-generating rather than confirmatory. Lastly, several secondary analyses yielded statistically significant results with small effect sizes and wide confidence intervals; statistical significance should not be equated with clinical relevance, and the primary paired differences and incremental in vitro coverage are, therefore, reported with confidence intervals to emphasise precision rather than relying on *p*-values alone.

## 6. Conclusions

Among the species for which EUCAST publishes clinical breakpoints for both agents, cefiderocol demonstrated consistently greater in vitro activity than colistin and retained activity against approximately three-quarters of the isolates resistant to colistin, an observation that is directionally consistent, though not identical in magnitude, in the wider collection. The limited concordance between the two agents supports paired susceptibility testing rather than assuming cross-resistance. Because no treatment, exposure or outcome data were available, these findings support paired in vitro susceptibility testing of cefiderocol and colistin as a diagnostic strategy that identifies isolates retaining cefiderocol activity when colistin does not; they do not establish that cefiderocol improves clinical outcomes. Therapeutic positioning requires multicentre clinical validation with patient-level outcome data, alongside antimicrobial stewardship.

## Figures and Tables

**Table 3:** Quality-control strains, media and acceptance ranges used for cefiderocol and colistin broth microdilution.

Agent	Quality-Control Strain	Medium	Acceptance Range (mg/L)	Modal MIC Observed (mg/L)	Determinations Within Range, n/N (%)
Cefiderocol	*E. coli* ATCC 25922	ID-CAMHB	0.03–0.25	0.06	46/48 (95.8)
Cefiderocol	*P. aeruginosa* ATCC 27853	ID-CAMHB	0.06–0.5	0.25	47/48 (97.9)
Colistin	*E. coli* ATCC 25922	CAMHB	0.25–2	0.5	48/48 (100)
Colistin	*P. aeruginosa* ATCC 27853	CAMHB	0.5–4	1	47/48 (97.9)
Colistin	*E. coli* NCTC 13846 (*mcr-1*-positive)	CAMHB	2–4 (expected MIC 4)	4	48/48 (100)
Total	—	—	—	—	236/240 (98.3)

ID-CAMHB, iron-depleted cation-adjusted Mueller–Hinton broth; CAMHB, cation-adjusted Mueller–Hinton broth. Acceptance ranges are those published by EUCAST and CLSI for broth microdilution. The cefiderocol control ranges are the ranges specified for iron-depleted broth and served as the daily verification that iron depletion was adequate. *E. coli* NCTC 13846 carries *mcr-1* and is the control strain EUCAST specifically requires for colistin broth microdilution. Determinations were made on each of 48 testing days for each strain–agent combination. MIC, minimum inhibitory concentration.

**Table 6:** Cefiderocol and colistin susceptibility stratified by bacterial species.

Species	n	Cefiderocol-S or I, n (%)	95% CI	Colistin-S, n/N (%)	95% CI
*K. pneumoniae*	121	97 (80.2)	72.2–86.3	38/106 (35.8)	27.4–45.3
*A. baumannii*	83	71 (85.5) ^‡^	76.4–91.5	67/82 (81.7)	72.0–88.6
*P. aeruginosa*	25	21 (84.0)	65.3–93.6	15/18 (83.3)	60.8–94.2
*E. coli*	4	2 (50.0)	15.0–85.0	2/2 (100)	34.2–100
*E. vulneris*	3	3 (100) ^‡^	43.8–100	3/3 (100)	43.8–100
Overall	236	194 (82.2)	76.8–86.6	125/211 (59.2)	52.5–65.6

Confidence intervals calculated by the Wilson score method. χ^2^ across five species for cefiderocol non-resistance: χ^2^ = 4.52, df = 4, *p* = 0.341. ^‡^ Cefiderocol results for *A. baumannii* and *E. vulneris* are counts of isolates inhibited at the PK/PD (non-species-related) value of 2 mg/L, not susceptibility categories: EUCAST publishes no clinical cefiderocol breakpoint for *A. baumannii* and no species-specific breakpoint for *E. vulneris*. Their MIC distributions are given in Section 3.4, and the primary analysis, which excludes them, in Table 5. The cefiderocol column combines the susceptible (S) and susceptible-at-increased-exposure (I) categories; the eight I isolates were all *K. pneumoniae* with an MIC of 4 mg/L, and the susceptible-only proportion for that species was 73.6% (95% CI 65.1–80.6). Colistin for *P. aeruginosa* was interpreted at the EUCAST parenthetical breakpoint of ≤4 mg/L; at the ≤2 mg/L value used for the other species the figure would be 61.1% (11/18). Fisher exact, *K. pneumoniae* versus *A. baumannii*: OR = 0.68, *p* = 0.355. Abbreviations: S, susceptible; CI, confidence interval.

**Table 7:** Cefiderocol non-resistance (susceptible or susceptible at increased exposure) stratified by specimen type.

Specimen Source	n	Cefiderocol-S or I, n (%)	95% CI (Wilson)
Urine	45	39 (86.7)	73.8–93.7
Purulent secretions	30	26 (86.7)	70.3–94.7
Blood	10	8 (80.0)	49.0–94.3
Respiratory	121	99 (81.8)	74.0–87.7
Catheter	30	22 (73.3)	55.6–85.8
Overall	236	194 (82.2)	76.8–86.6

χ^2^ across specimen types: χ^2^ = 2.68, df = 4, *p* = 0.613. Cefiderocol-S or I combines the susceptible and susceptible-at-increased-exposure categories, and the counts include *A. baumannii* and *E. vulneris* isolates classified descriptively against the PK/PD value of 2 mg/L. Abbreviations: S, susceptible; CI, confidence interval.

**Table 8:** Secondary descriptive analysis: paired cross-tabulation of cefiderocol and colistin phenotypes and agreement statistics across the full collection (n = 211 isolates with both results available).

	Colistin-S	Colistin-R	Row Total
Cefiderocol-S or I	104	67	171 (81.0%)
Cefiderocol-R	21	19	40 (19.0%)
Column total	125 (59.2%)	86 (40.8%)	211
Agreement statistic	Value	95% CI	*p*-value
Observed (raw) agreement	58.3% (123/211)	—	—
Cohen’s κ	0.058	−0.093 to 0.208	—
McNemar χ^2^ (continuity-corrected)	23.01	—	< 0.001
McNemar exact (binomial)	—	—	< 0.001

Abbreviations: S, susceptible; R, resistant; CI, confidence interval. I, susceptible at increased exposure. This is a secondary descriptive cross-tabulation of the full collection; cefiderocol categories for *A. baumannii* and *E. vulneris* are PK/PD-based and not clinical categories. The primary cross-tabulation, restricted to species–agent combinations with EUCAST clinical breakpoints, is given in Table 5.

**Table 9:** MIC_50_, MIC_90_, range, and geometric mean MIC by species and agent.

Species	Agent	n	MIC Range (µg/mL)	MIC_50_	MIC_90_	Geometric Mean
*K. pneumoniae*	Cefiderocol	121	0.06–32	1	8	1.39
*K. pneumoniae*	Colistin	104	0.5–32	8	16	4.36
*A. baumannii*	Cefiderocol	82	0.125–16	1	4	0.90
*A. baumannii*	Colistin	79	0.25–32	1	4	1.12
*P. aeruginosa*	Cefiderocol	25	0.125–8	0.5	8	1.00
*P. aeruginosa*	Colistin	18	0.5–8	1	8	2.00

Wilcoxon signed-rank test comparing paired cefiderocol and colistin MICs across all isolates with both values (n = 206): W = 3078, *p* < 0.001. Abbreviation: MIC, minimum inhibitory concentration. MIC distributions are reported unchanged from the original submission; only their categorical interpretation has been revised. The breakpoints applied to each species–agent combination are given in Table 2.

**Table 10:** Conditional in vitro susceptibility of cefiderocol among colistin-resistant isolates.

Subgroup	Colistin-R Isolates (n)	Cefiderocol-S or I Among Them, n (%)	95% CI (Wilson)
*K. pneumoniae*	68	51 (75.0)	63.6–83.8
*A. baumannii*	15	13 (86.7) ^‡^	62.1–96.3
*P. aeruginosa*	3	3 (100)	43.9–100
Overall (primary analysis, breakpoint-valid species)	71	54 (76.1)	65.0–84.5
Overall (secondary, full collection)	86	67 (77.9)	68.1–85.4

Conditional in vitro susceptibility = the probability that an isolate is cefiderocol-susceptible in vitro given that it is colistin-resistant. It is a laboratory descriptor only and does not denote demonstrated clinical benefit; no treatment or outcome data were available in this study. Abbreviations: R, resistant; S, susceptible; CI, confidence interval. I, susceptible at increased exposure. Cefiderocol-S or I combines the susceptible and susceptible-at-increased-exposure categories. ^‡^ EUCAST publishes no clinical cefiderocol breakpoint for *A. baumannii*; the *A. baumannii* row counts colistin-resistant isolates with a cefiderocol MIC ≤ 2 mg/L and is descriptive only, which is why it contributes to the secondary but not the primary overall estimate. Colistin resistance for *P. aeruginosa* is defined by the EUCAST parenthetical breakpoint of >4 mg/L; at the >2 mg/L value used for the other species the subgroup would contain seven isolates, all cefiderocol-non-resistant.

**Table 11:** Multivariable logistic regression—predictors of cefiderocol resistance in the primary breakpoint-valid paired population (n = 126) and in the full paired collection (n = 211).

Predictor	Adjusted OR	95% CI	*p*-Value
Primary model: breakpoint-valid paired population (n = 126; 29 events)	—	—	—
Age (per 10-year increase)	0.71	0.52–0.97	0.032
Female sex (versus male)	0.94	0.40–2.21	0.888
*E. coli* (versus *K. pneumoniae*)	6.42	0.54–76.34	0.142
*P. aeruginosa* (versus *K. pneumoniae*)	0.79	0.20–3.12	0.740
Colistin resistance (versus susceptible)	1.24	0.51–3.02	0.639
Model McFadden pseudo-R^2^	0.061	—	—
Secondary model: full paired collection (n = 211; 40 events)	—	—	—
Age (per 10-year increase)	0.68	0.51–0.90	0.007
Female sex (versus male)	0.92	0.44–1.94	0.828
*A. baumannii* (versus *K. pneumoniae*)	0.52	0.22–1.24	0.140
*P. aeruginosa* (versus *K. pneumoniae*)	0.49	0.13–1.87	0.297
Other species (versus *K. pneumoniae*)	2.61	0.36–18.94	0.343
Colistin resistance (versus susceptible)	1.19	0.54–2.61	0.663
Model McFadden pseudo-R^2^	0.072	—	—

Reference category for species is *K. pneumoniae*. Outcome modelled is cefiderocol resistance. Abbreviations: OR, odds ratio; CI, confidence interval. The primary model is fitted to the breakpoint-valid paired population (*K. pneumoniae*, *E. coli*, *P. aeruginosa*), in which cefiderocol resistance is defined by the species-specific EUCAST v16.1 breakpoints of Table 2 and isolates at 4 mg/L are classified as susceptible at increased exposure rather than resistant. The secondary model is fitted to the full paired collection and assigns cefiderocol categories to *A. baumannii* and *E. vulneris* on a PK/PD basis; its species coefficients must be read with that limitation in mind. Refitting the primary model with cluster-robust standard errors clustered on patient changed no estimate materially. Both models are exploratory.

**Table 12:** MIC-level concordance, systematic bias, and correlation between cefiderocol and colistin (n = 206 paired MIC values).

Metric	Value	Interpretation
Spearman ρ (log_2_ MIC, cefiderocol versus colistin)	0.196 (*p* = 0.005)	Weak positive rank correlation
Median cefiderocol MIC (µg/mL)	1.0	—
Median colistin MIC (µg/mL)	1.0	—
Mean log_2_ difference (colistin − cefiderocol)	+0.90	Colistin MICs ≈ 1.87-fold higher
Wilcoxon signed-rank (paired MICs)	W = 3078, *p* < 0.001	Significant systematic shift
Cohen’s κ (categorical, from Table 5 and Table 8)	0.020 (primary); 0.058 (secondary)	Negligible agreement

Abbreviation: MIC, minimum inhibitory concentration.

**Table 13:** Incremental in vitro coverage, coverage gain, and incremental diagnostic yield in the primary breakpoint-valid population (n = 126 paired isolates) and in the full collection (n = 211 paired isolates).

Population	Colistin Alone, n (%)	Cefiderocol Alone, n (%)	Either Agent, n (%)	Net Cefiderocol Gain
Primary analysis, breakpoint-valid (n = 126)	55 (43.7%)	97 (77.0%)	109 (86.5%)	+33.3 pts; 1 per 2.3 matched isolates
Secondary, full collection (n = 211)	125 (59.2%)	171 (81.0%)	192 (91.0%)	+21.8 pts; 1 per 3.1 matched isolates
*K. pneumoniae* (n = 106)	—	—	—	Gain 48.1%; reverse 6.6%; net +41.5%
*A. baumannii* (n = 82)	—	—	—	Gain 15.9%; reverse 11.0%; net +4.9%
*P. aeruginosa* (n = 18)	—	—	—	Gain 16.7%; reverse 16.7%; net 0.0%

“Gain” = colistin-R but cefiderocol-S. “Reverse” = colistin-S but cefiderocol-R. Incremental diagnostic yield = the number of isolates that must undergo paired testing to identify one additional colistin-resistant isolate that remains cefiderocol-non-resistant in vitro. Its denominator is all matched isolates, that is every isolate with an interpretable result for both agents (126 in the primary population, 211 in the full collection), and not the colistin-resistant subgroup; it is the reciprocal of 42.9% and 31.8% respectively. It is a diagnostic, not a therapeutic, measure. Species rows are drawn from the full collection; the *A. baumannii* row is descriptive only because that species has no clinical cefiderocol breakpoint. Abbreviations: pts, percentage points; R, resistant; S, susceptible.

**Table 14:** Cefiderocol MIC distributions for the species that have no EUCAST clinical cefiderocol breakpoint, reported without categorical assignment and set against the EUCAST evidence tiers.

MIC (µg/mL)	*A. baumannii*, n (%) (n = 82)	Cumulative %	*E. vulneris*, n (%) (n = 3)	Cumulative %
0.06	0 (0)	0	0 (0)	0
0.125	6 (7.3)	7.3	0 (0)	0
0.25	0 (0)	7.3	1 (33.3)	33.3
0.5	32 (39.0)	46.3	1 (33.3)	66.7
1	29 (35.4)	81.7	1 (33.3)	100
2	3 (3.7)	85.4	0 (0)	100
4	4 (4.9)	90.2	0 (0)	100
8	5 (6.1)	96.3	0 (0)	100
16	3 (3.7)	100	0 (0)	100
32	0 (0)	100	0 (0)	100
EUCAST evidence tier ≤ 0.5 mg/L (evidence sufficient)	38 (46.3)	46.3	2 (66.7)	66.7
EUCAST evidence tier 1–2 mg/L (evidence insufficient)	32 (39.0)	85.4	1 (33.3)	100
EUCAST evidence tier > 2 mg/L (response unlikely)	12 (14.6)	100	0 (0)	100
MIC_50_ (µg/mL)	1	—	0.5	—
MIC_90_ (µg/mL)	4	—	1	—
MIC range (µg/mL)	0.125–16	—	0.25–1	—
Geometric mean MIC (µg/mL)	0.90	—	0.50	—

Distributions are shown for the isolates with an interpretable cefiderocol MIC (*A. baumannii* n = 82 of 83; *E. vulneris* n = 3 of 3). EUCAST does not publish a clinical cefiderocol breakpoint for *A. baumannii* or a species-specific breakpoint for *E. vulneris*, so no susceptible or resistant category is assigned here. For *A. baumannii*, EUCAST instead grades the MIC into three bands carrying different levels of evidence—≤0.5 mg/L, 1–2 mg/L and >2 mg/L—and the corresponding proportions are given in the three evidence-tier rows; these are statements about the strength of the supporting evidence, not susceptibility categories, and no S/I/R interpretation should be derived from them. The PK/PD (non-species-related) value of 2 mg/L used descriptively in the secondary analyses corresponds to a cumulative 85.4% for *A. baumannii* and 100% for *E. vulneris*. Abbreviation: MIC, minimum inhibitory concentration.

**Table 15:** Primary analysis, restricted to species–agent combinations with EUCAST species-specific clinical breakpoints for both agents, set against the secondary descriptive analysis of the full collection.

Metric	Primary Analysis (Breakpoint-Valid Species)	Secondary Descriptive Analysis (Full Collection)
Isolates included, n	150 (*K. pneumoniae* 121, *E. coli* 4, *P. aeruginosa* 25)	236 (all species)
Isolates with paired colistin MIC, n	126	211
Cefiderocol non-resistant (S or I), n/N (%) [95% CI]	120/150 (80.0) [72.9–85.6]	194/236 (82.2) [76.8–86.6]
Cefiderocol susceptible (S) only, n/N (%) [95% CI]	112/150 (74.7) [67.2–81.0]	186/236 (78.8) [73.2–83.5]
Cefiderocol non-resistant, paired subset, n/N (%) [95% CI]	97/126 (77.0) [68.9–83.5]	171/211 (81.0) [75.2–85.8]
Colistin-susceptible, paired subset, n/N (%) [95% CI]	55/126 (43.7) [35.3–52.4]	125/211 (59.2) [52.5–65.6]
Cefiderocol S or I/colistin-S, n	43	104
Cefiderocol S or I/colistin-R, n	54	67
Cefiderocol-R/colistin-S, n	12	21
Cefiderocol-R/colistin-R, n	17	19
Observed (raw) agreement	47.6% (60/126)	58.3% (123/211)
Cohen’s κ (95% CI)	0.020 (−0.144 to 0.183)	0.058 (−0.093 to 0.208)
McNemar χ^2^ (continuity-corrected), *p*	25.47, *p* < 0.001	23.01, *p* < 0.001
Colistin-resistant isolates, n	71	86
Conditional in vitro susceptibility to cefiderocol, n/N (%) [95% CI]	54/71 (76.1) [65.0–84.5]	67/86 (77.9) [68.1–85.4]
Coverage by either agent, n/N (%) [95% CI]	109/126 (86.5) [79.5–91.4]	192/211 (91.0) [86.4–94.2]
Incremental in vitro coverage (cefiderocol over colistin)	+33.3 percentage points	+21.8 percentage points
Incremental diagnostic yield	1 additional isolate per 2.3 matched isolates tested	1 additional isolate per 3.1 matched isolates tested

The breakpoint-valid subpopulation comprises the species for which EUCAST publishes species-specific clinical breakpoints for both cefiderocol and colistin. *A. baumannii* (n = 83), for which no species-specific cefiderocol breakpoint exists, and *E. vulneris* (n = 3), for which no species-specific breakpoint exists for either agent, are excluded and are presented as MIC distributions in Table 14. Confidence intervals were calculated by the Wilson score method. Conditional in vitro susceptibility denotes the probability of cefiderocol susceptibility in vitro given colistin resistance and does not denote clinical benefit. Abbreviations: S, susceptible; R, resistant; MIC, minimum inhibitory concentration; CI, confidence interval. I, susceptible at increased exposure. The primary column is the analysis on which the study’s conclusions rest; the secondary column assigns cefiderocol categories to *A. baumannii* and *E. vulneris* against the PK/PD value of 2 mg/L and is descriptive only. Cefiderocol non-resistant combines the S and I categories, the S-only figures being given in the row above. Colistin for *P. aeruginosa* is interpreted at the EUCAST parenthetical breakpoint of ≤4 mg/L. The incremental diagnostic yield has all matched isolates as its denominator.

**Table 16:** Patient-level sensitivity analysis: primary paired results recomputed after restricting the dataset to the first isolate obtained from each patient.

Metric	Primary Analysis (All Breakpoint-Valid Isolates)	Patient-Level Sensitivity Analysis (First Isolate Per Patient)
Unique patients contributing, n	220	220
Isolates included, n	150	139
Isolates with a paired colistin MIC, n	126	117
Cefiderocol non-resistant, paired subset, n/N (%) [95% CI]	97/126 (77.0) [68.9–83.5]	90/117 (76.9) [68.5–83.6]
Colistin susceptible, paired subset, n/N (%) [95% CI]	55/126 (43.7) [35.3–52.4]	52/117 (44.4) [35.8–53.5]
Discordant cells (cefiderocol non-R/colistin-R: cefiderocol-R/colistin-S)	54:12	49:11
Cohen’s κ (95% CI)	0.020 (−0.144 to 0.183)	0.032 (−0.139 to 0.203)
McNemar χ^2^ (continuity-corrected), *p*	25.47, *p* < 0.001	22.82, *p* < 0.001
Conditional in vitro susceptibility, n/N (%) [95% CI]	54/71 (76.1) [65.0–84.5]	49/65 (75.4) [63.7–84.2]
Incremental in vitro coverage	+33.3 percentage points	+32.5 percentage points
Incremental diagnostic yield	1 per 2.3 matched isolates	1 per 2.4 matched isolates

The 236 isolates were obtained from 220 unique patients; de-duplication was applied per species, so 14 patients contributed two isolates of different species and one contributed three. The patient-level column retains only the first isolate obtained from each patient, restoring strict independence between observations. Confidence intervals are Wilson score intervals. Cefiderocol non-resistant combines the susceptible and susceptible-at-increased-exposure categories. R, resistant; S, susceptible; CI, confidence interval; MIC, minimum inhibitory concentration.

## Data Availability

The data supporting the findings of this study are available from the corresponding authors upon reasonable request.

## Supplementary Materials

The following supporting information can be downloaded at: https://www.mdpi.com/article/10.3390/microorganisms14092071/s1, Table S1: Species-stratified antimicrobial categories, denominators and non-susceptibility frequencies used to apply the extensively drug-resistant (XDR) definition (n = 236); Table S2: Antimicrobial agents, test methods and breakpoint sources used to apply the extensively drug-resistant (XDR) definition, by species.

## Abbreviations

The following abbreviations are used in this manuscript:

XDR	Extensively drug-resistant
MIC	Minimum inhibitory concentration
EUCAST	European Committee on Antimicrobial Susceptibility Testing
CI	Confidence interval
OR	Odds ratio
IDY	Incremental diagnostic yield
I	Susceptible, increased exposure
QC	Quality control
ID-CAMHB	Iron-depleted cation-adjusted Mueller–Hinton broth
S/R	Susceptible/resistant
