# Supplementary material for: Paired In Vitro Susceptibility of Cefiderocol and Colistin in Colistin-Resistant, Extensively Drug-Resistant Gram-Negative Bacilli: A Single-Centre, MIC-Based Analysis of 236 Clinical Isolates
Source: Microorganisms. 2026 Sep 16;14(9):2071. doi: 10.3390/microorganisms14092071 (PMC13609237; doi:10.3390/microorganisms14092071)
Supplement: Supplementary file 1 [file microorganisms-14-02071-s001.zip › microorganisms-4535786-supplementary.pdf]

Table S1. Species-stratified antimicrobial categories, denominators and non-susceptibility frequencies used to apply the extensively drug-resistant (XDR) definition (n = 236).

**Antimicrobial category**	***K. pneumoniae* (n = 121)**	***A. baumannii* (n = 83)**	***P. aeruginosa* (n = 25)**	***Escherichia* spp. (n = 7)**
Antipseudomonal carbapenems	121/121 (100)	83/83 (100)	25/25 (100)	7/7 (100)
Extended-spectrum cephalosporins	121/121 (100)	83/83 (100) ‡	25/25 (100)	5/7 (71.4)
Antipseudomonal fluoroquinolones	118/121 (97.5)	82/83 (98.8)	24/25 (96.0)	4/7 (57.1)
Antipseudomonal penicillins + BLI	120/121 (99.2)	82/83 (98.8) ‡	24/25 (96.0)	5/7 (71.4)
Aminoglycosides	110/121 (90.9)	76/83 (91.6)	20/25 (80.0)	4/7 (57.1)
Folate-pathway inhibitors	108/121 (89.3)	74/83 (89.2)	19/25 (76.0) ‡	4/7 (57.1)
Monobactam / BLI combination	98/121 (81.0)	83/83 (100) ‡	24/25 (96.0) ‡	4/7 (57.1)
Cephalosporin / BLI combination	85/121 (70.2)	83/83 (100) ‡	18/25 (72.0)	4/7 (57.1)
Glycylcyclines / tetracyclines	65/121 (53.7)	58/83 (69.9) ‡	25/25 (100) ‡	2/7 (28.6)
Polymyxins (colistin) §	68/106 (64.2)	15/82 (18.3)	3/18 (16.7)	0/5 (0)
Siderophore cephalosporin (cefiderocol) §	24/121 (19.8)	12/83 (14.5)	4/25 (16.0)	2/7 (28.6)
**Categories scored towards the XDR definition, n**	**9**	**4**	**6**	**9**

Values are the number of isolates of that species non-susceptible to at least one representative agent in the category, over the number tested; row totals reproduce the aggregate figures given in Table 1. ‡ Species–agent combination affected by intrinsic resistance or by the absence of a EUCAST clinical breakpoint for that species: extended-spectrum cephalosporins, piperacillin-tazobactam, tigecycline, ceftazidime-avibactam and aztreonam-avibactam for A. baumannii, and trimethoprim-sulfamethoxazole, tigecycline and aztreonam-avibactam for P. aeruginosa. This list has been corrected in the present version: EUCAST v16.1 publishes no breakpoints for A. baumannii for extended-spectrum cephalosporins, piperacillin-tazobactam or tigecycline, so these three categories, which were previously scored, have been removed from the XDR calculation for that species, reducing its scored categories from seven to four (carbapenems, fluoroquinolones, aminoglycosides and folate-pathway inhibitors). Results for these combinations are shown for completeness but were not scored as acquired non-susceptibility when the XDR definition was applied, which is why the number of categories contributing to the definition differs by species. § Colistin and cefiderocol are the two study agents; their results are shown for completeness and were not counted towards the XDR definition, and the colistin denominators exclude isolates without an interpretable colistin MIC. *Escherichia* spp. comprises *E. coli* (n = 4) and *E. vulneris* (n = 3), presented together because of small numbers. BLI, beta-lactamase inhibitor. All 83 A. baumannii isolates continue to satisfy the XDR definition on the four EUCAST-breakpointed categories alone, so the correction changed no isolate's inclusion status; it does, however, mean that the XDR label is less discriminating in this species than in the others, and the category-level frequencies should be read alongside it. Colistin non-susceptibility for P. aeruginosa and cefiderocol non-susceptibility for K. pneumoniae have likewise been recomputed against the corrected species-specific breakpoints of Table 2. The agents tested, the test method used for each and the breakpoint source applied are given in Table S2.

Table S2. Antimicrobial agents, test methods and breakpoint sources used to apply the extensively drug-resistant (XDR) definition, by species.

**Antimicrobial category**	**Agent(s) tested**	**Test method**	***K. pneumoniae / E. coli / E. vulneris***	***A. baumannii***	***P. aeruginosa***
Antipseudomonal carbapenems	Meropenem, imipenem	Disc diffusion, MIC confirmation where zone was near the breakpoint	EUCAST v16.1 — scored	EUCAST v16.1 — scored	EUCAST v16.1 — scored
Extended-spectrum cephalosporins	Ceftazidime, cefepime	Disc diffusion	EUCAST v16.1 — scored	No EUCAST breakpoint — not scored	EUCAST v16.1 — scored
Antipseudomonal fluoroquinolones	Ciprofloxacin, levofloxacin	Disc diffusion	EUCAST v16.1 — scored	EUCAST v16.1 — scored	EUCAST v16.1 — scored
Antipseudomonal penicillins + BLI	Piperacillin-tazobactam	Disc diffusion	EUCAST v16.1 — scored	No EUCAST breakpoint — not scored	EUCAST v16.1 — scored
Aminoglycosides	Gentamicin, amikacin, tobramycin	Disc diffusion	EUCAST v16.1 — scored	EUCAST v16.1 — scored	EUCAST v16.1 (amikacin, tobramycin) — scored
Folate-pathway inhibitors	Trimethoprim-sulfamethoxazole	Disc diffusion	EUCAST v16.1 — scored	EUCAST v16.1 — scored	Intrinsic resistance — not scored
Monobactam / BLI combination	Aztreonam-avibactam	Disc diffusion	EUCAST v16.1 — scored	No EUCAST breakpoint — not scored	No EUCAST breakpoint — not scored
Cephalosporin / BLI combination	Ceftazidime-avibactam	Disc diffusion	EUCAST v16.1 — scored	No EUCAST breakpoint — not scored	EUCAST v16.1 — scored
Glycylcyclines / tetracyclines	Tigecycline	Broth microdilution	EUCAST v16.1 — scored	No EUCAST breakpoint — not scored	Intrinsic resistance — not scored
Polymyxins (study agent)	Colistin sulfate	Reference broth microdilution (UMIC, CAMHB)	EUCAST v16.1, S ≤ 2 mg/L — not counted towards XDR	EUCAST v16.1, S ≤ 2 mg/L — not counted towards XDR	EUCAST v16.1 parenthetical, S ≤ 4 mg/L — not counted towards XDR
Siderophore cephalosporin (study agent)	Cefiderocol	Reference broth microdilution in ID-CAMHB (UMIC)	EUCAST v16.1, S ≤ 2 / I 4 / R > 4 mg/L — not counted towards XDR	No clinical breakpoint; MIC distribution only — not counted towards XDR	EUCAST v16.1, S ≤ 2 / R > 2 mg/L — not counted towards XDR
**Categories scored towards the XDR definition, n**	—`	—	**9**	**4**	**6**

All categorical interpretations were made with EUCAST Breakpoint Table v16.1 (2026). A category was scored towards the XDR definition for a given species only where EUCAST publishes a clinical breakpoint for that species–agent combination and the species is not intrinsically resistant; combinations marked as not scored were tested and reported for completeness but contributed nothing to the classification of any isolate. Disc diffusion was used for the screening categories, with broth-microdilution MIC confirmation for isolates whose zone diameters fell within one millimetre of a breakpoint; the two study agents were determined by reference broth microdilution throughout, and neither was counted towards the XDR definition. Because only four categories retain EUCAST breakpoints for A. baumannii once the study agents are excluded, the XDR definition is less discriminating in that species; all 83 A. baumannii isolates nonetheless met the definition on those four categories. BLI, beta-lactamase inhibitor; CAMHB, cation-adjusted Mueller–Hinton broth; ID-CAMHB, iron-depleted cation-adjusted Mueller–Hinton broth; MIC, minimum inhibitory concentration; S, susceptible; I, susceptible at increased exposure; R, resistant.
